# Supplementary material for: Fall-related measures in elderly individuals and Parkinson’s disease subjects
Source: PLoS One. 2020 Aug 13;15(8):e0236886. doi: 10.1371/journal.pone.0236886 (PMC7425912; doi:10.1371/journal.pone.0236886)
Supplement: Attachment — Submitted filename: PONE-D-20-08972_reviewer comments.docx [file pone.0236886.s002.docx]

PLOS ONE

Predictive measures of falls in elderly and Parkinson’s Disease

--Manuscript Draft--

| Manuscript Number: | PONE-D-20-08972 |
| --- | --- |
| Article Type: | Research Article |
| Full Title: | Predictive measures of falls in elderly and Parkinson’s Disease |
| Short Title: | Falls in elderly and Parkinson's Disease |
| Corresponding Author: | Justyna Michalska, PhD  The Jerzy Kukuczka Acadamy of Physical Education Katowice, POLAND |
| Keywords: | limit of stability test, fear of falling, safety margin, postural control, balance clinical test |
| Abstract: | Falls pose a serious problem in elderly and clinical populations. Most often they lead to a loss of mobility and independence. They might be as well an indirect cause of death. The aim of this study was to determine predictive measures of fall in elderly subjects and Parkinson’s Disease patients. Thirty-two elderly subjects were examined in this study from which sixteen were diagnosed with PD. The testing procedures comprised of force plate measurements (limit of stability test–LOS test) and clinical tests: Berg Balance Scale, Functional Reach Test, Time Up and GO, Tinetti Test. The Falls Efficacy Scale International (FES-I) was used to evaluate fear of falling. The range of maximum forward lean was normalized to length from the ankle joint to the head of first metatarsal bone and it was named functional forward stability indicator (FFSI). The FFSI derived from the LOS test, allowed to demonstrate the real deficit in functional stability and individual safety margins. Moreover FFSI highly correlated with FES-I and all clinical tests in elderly subjects (r>0,6; p<0,05). In PD FFSI poorly correlated with fear of falling, Berg Balance Scale and Functional Reach test, however, a high correlation with the number the falls (r>0,6, p<0,05) was noted. The PD subjects presented a different balance strategy when closing to their stability limits which was also reflected in the lower values of sample entropy (t= (-2,40); p>0.05; d=0,87). FFSI occurred to be a good predictor of fear of falling in the group of elderly people. Additionally, it allows to show real balance deficits both in PD and their healthy peers without the need for a reference group and norms. In conclusion, it is postulated that the popular clinical assessment of postural balance in PD should be accompanied by reliable posturography measurements. |
| Order of Authors: | Justyna Michalska, PhD |
|  | Anna Kamieniarz |
|  | Anna Brachman |
|  | Wojciech Marszałek |
|  | Joanna Cholewa |
|  | Grzegorz Juras |
|  | Kajetan J. Słomka |
| Additional Information: |  |
| Question | Response |
| Financial Disclosure  Enter a financial disclosure statement that describes the sources of funding for the work included in this submission. Review | The study was supported by the National Center for Research and Development Grant under the program STRATEG MED III within the “VB-Clinic” project no.  STRATEGMED3/306011/1/NCBR/2017. |

| the [submission guidelines](http://journals.plos.org/plosone/s/submission-guidelines#loc-financial-disclosure-statement) for detailed requirements. View published research articles from [PLOS ONE](http://journals.plos.org/plosone/) for specific examples.  This statement is required for submission and will appear in the published article if the submission is accepted. Please make sure it is accurate. |  |
| --- | --- |
| Unfunded studies Enter: The author(s) received no specific funding for this work. Funded studies Enter a statement with the following details: - Initials of the authors who received each award - Grant numbers awarded to each author - The full name of each funder - URL of each funder website - Did the sponsors or funders play any role in the study design, data collection and analysis, decision to publish, or preparation of the manuscript? - NO - Include this sentence at the end of your statement: The funders had no role in study design, data collection and analysis, decision to publish, or preparation of the manuscript. - YES - Specify the role(s) played. |  |
| * typeset |  |
| Competing Interests  Use the instructions below to enter a competing interest statement for this submission. On behalf of all authors, disclose any [competing interests](http://journals.plos.org/plosone/s/competing-interests) that could be perceived to bias this work—acknowledging all financial support and any other relevant financial or nonfinancial competing interests.  This statement will appear in the published article if the submission is accepted. Please make sure it is accurate. View published research articles from [PLOS ONE](http://journals.plos.org/plosone/) for specific examples. | The authors have declared that no competing interests exist. |

| NO authors have competing interests Enter: The authors have declared that no competing interests exist. Authors with competing interests Enter competing interest details beginning with this statement: I have read the journal's policy and the authors of this manuscript have the following competing interests: [insert competing interests here] |  |
| --- | --- |
| * typeset |  |
| Ethics Statement  Enter an ethics statement for this submission. This statement is required if the study involved:   - Human participants - Human specimens or tissue - Vertebrate animals or cephalopods - Vertebrate embryos or tissues - Field research   Write "N/A" if the submission does not require an ethics statement.  General guidance is provided below. Consult the [submission guidelines](http://journals.plos.org/plosone/s/submission-guidelines#loc-guidelines-for-specific-study-types) for detailed instructions. Make sure that all information entered here is included in the Methods section of the manuscript. | The research was accepted by the Institutional Ethics Committee Medical University in Warsaw number KB/28/2014 |

Format for specific study types

Human Subject Research (involving human

participants and/or tissue)

Give the name of the institutional review

board or ethics committee that approved the

study

•

Include the approval number and/or a

statement indicating approval of this

research

•

Indicate the form of consent obtained

(

written/oral) or the reason that consent was

not obtained (e.g. the data were analyzed

anonymously)

•

Animal Research (involving vertebrate

animals, embryos or tissues)

Provide the name of the Institutional Animal

Care and Use Committee (IACUC) or other

relevant ethics board that reviewed the

study protocol, and indicate whether they

approved this research or granted a formal

waiver of ethical approval

•

Include an approval number if one was

obtained

•

If the study involved

non-human primates

,

add

additional details

about animal welfare

and steps taken to ameliorate suffering

•

If anesthesia, euthanasia, or any kind of

animal sacrifice is part of the study, include

briefly which substances and/or methods

were applied

•

Field Research

Include the following details if this study

involves the collection of plant, animal, or

other materials from a natural setting:

Field permit number

•

Name of the institution or relevant body that

granted permission

•

Data Availability

Authors are required to make all data

underlying the findings described fully

available, without restriction, and from the

time of publication. PLOS allows rare

exceptions to address legal and ethical

concerns. See the

[PLOS Data Polic](http://journals.plos.org/plosone/s/data-availability)

[y](http://journals.plos.org/plosone/s/data-availability)

and

[FA](http://journals.plos.org/plosone/s/data-availability#loc-faqs-for-data-policy)

[Q](http://journals.plos.org/plosone/s/data-availability#loc-faqs-for-data-policy)

for detailed information.

Yes - all data are fully available without restriction

| A Data Availability Statement describing where the data can be found is required at submission. Your answers to this question constitute the Data Availability Statement and will be published in the article, if accepted. |  |
| --- | --- |
| Important: Stating ‘data available on request from the author’ is not sufficient. If your data are only available upon request, select ‘No’ for the first question and explain your exceptional situation in the text box. |  |
| Do the authors confirm that all data underlying the findings described in their manuscript are fully available without restriction? |  |
| Describe where the data may be found in full sentences. If you are copying our sample text, replace any instances of XXX with the appropriate details.   - If the data are held or will be held in a public repository, include URLs, accession numbers or DOIs. If this information will only be available after acceptance, indicate this by ticking the box below. For example: All XXX files   are available from the XXX database (accession number(s) XXX, XXX.).   - If the data are all contained within the manuscript and/or Supporting Information files, enter the following: All relevant data are within the manuscript and its Supporting Information files. - If neither of these applies but you are able to provide details of access elsewhere, with or without limitations, please do so. For example:   Data cannot be shared publicly because of [XXX]. Data are available from the XXX Institutional Data Access / Ethics Committee (contact via XXX) for researchers who meet the criteria for access to confidential data.  The data underlying the results presented in the study are available from (include the name of the third party | All relevant data are within the manuscript and its Supporting Information files. |

| and contact information or URL).  • This text is appropriate if the data are owned by a third party and authors do not have permission to share the data.  * typeset |  |
| --- | --- |
| Additional data availability information: |  |

Manuscript	[Click here to download Manuscript manuscript.docx](https://www.editorialmanager.com/pone/download.aspx?id=26312209&guid=297aae80-8d01-4d36-96e9-fd6da5efd151&scheme=1)

# 1 Predictive measures of falls in elderly and Parkinson’s Disease

2	Justyna Michalska^1¶*^, Anna Kamieniarz^1¶^, Anna Brachman^1¶^, Wojciech Marszałek^1¶^, Joanna 3	Cholewa^2&^ Grzegorz Juras^1&^, Kajetan J. Słomka^1&^

4

5	^1^Institute of Sport Sciences, The Jerzy Kukuczka Academy of Physical Education, Katowice, 6	Poland

7	^2^ Department of Physiotherapy, The Jerzy Kukuczka Academy of Physical Education, 8	Katowice, Poland

9

10

11 *** Corresponding author:**
12 Justyna Michalska PhD
13 Institute of Sport Sciences
14 The Jerzy Kukuczka Academy of Physical Education
15 in Katowice, Poland
16 tel.: +4832 207 51 60
17 email: j.michalska@awf.katowice.pl

18

# Abstract

Falls pose a serious problem in elderly and clinical populations. Most often they lead to a loss of mobility and independence. They might be as well an indirect cause of death. The aim of this study was to determine predictive measures of fall in elderly subjects and Parkinson’s Disease patients. Thirty-two elderly subjects were examined in this study from which sixteen were diagnosed with PD. The testing procedures comprised of force plate measurements (limit of stability test–LOS test) and clinical tests: Berg Balance Scale, Functional Reach Test, Time Up and GO, Tinetti Test. The Falls Efficacy Scale International (FES-I) was used to evaluate fear of falling. The range of maximum forward lean was normalized to length from the ankle joint to the head of first metatarsal bone and it was named functional forward stability indicator (FFSI). The FFSI derived from the LOS test, allowed to demonstrate the real deficit in functional stability and individual safety margins. Moreover FFSI highly correlated with FESI and all clinical tests in elderly subjects (r>0,6; p<0,05). In PD FFSI poorly correlated with fear of falling, Berg Balance Scale and Functional Reach test, however, a high correlation with the number the falls (r>0,6, p<0,05) was noted. The PD subjects presented a different balance strategy when closing to their stability limits which was also reflected in the lower values of sample entropy (t= (-2,40); p>0.05; d=0,87). FFSI occurred to be a good predictor of fear of falling in the group of elderly people. Additionally, it allows to show real balance deficits both in PD and their healthy peers without the need for a reference group and norms. In conclusion, it is postulated that the popular clinical assessment of postural balance in PD should be accompanied by reliable posturography measurements.

# Introduction

Unintentional falls among people over 65 years old is an increasing problem, being the leading cause of injury-related deaths (1). According to WHO, between 2015 and 2050, the proportion of the world's population over 60 years old will nearly double from 12% to 22%. Currently, in the developed countries, the percentage of elderly subjects (ES) come to more than 20%. The ES experience falls due to significant deterioration of balance ability associated with decrease of muscle strength, sensory function and widespread degeneration in the central nervous system.

Moreover, with age, accompanying neurological diseases may appear. Parkinson’s Disease (PD) is the second most common neurodegenerative disorder (2). This chronic and progressive disease leads to rigidity, tremor, bradykinesia and thus impaired balance (3). So PD patients are more exposed to the risk of falls than their peers (4).

The fear of falling (FOF) could appear well before and/or after an incident of fall which creates a vicious cycle. FOF is related to the subjective body imagination and body efficiency. It might also be a proper reaction to certain situations, leading the elderly to be cautious behaviour, and can contribute to fall prevention through careful choice of movement activity. Nevertheless, ES increasingly cease physical activity because of the FOF (5).

FOF is assessed with many self-reported questionnaires among which Falls Efficacy Scale International test (FES-I) is widely used (6). It has become a useful instrument for predicting risk of falls in the elderly due to its excellent reliability and validity across different populations (7). The questionnaire refers to the most of the basic, everyday activities presented by disabled people would be likely to have difficulty with because of their FOF.

Most daily activities require keeping the center of gravity (COG) within the confines of the base of support (BOS). Therefore, the falls are quite frequent during everyday activities, especially when the COG is voluntarily shifted near or outside the limit of stability (8). Limit of stability is depicted by the stability boundary and anatomically was assumed to be the foot envelope. But the COG usually travels along a much smaller area of the BOS (9). It is suggested that the anterior mechanical stability boundary is located on the line connecting the extreme points located in the sagittal plane at the height of the first heads of the metatarsal bones (10). Moreover, from a biomechanical point of view, the rotation axis is located slightly further than the area of first metatarsophalangeal joint (11). Crossing this mechanical limit inevitably involves change of standing strategy (heel rise or toes co-contraction) or impose gait initiation. The most frequent measure used to identify stability boundary is limit of stability test (LOS test) (12). During this test a detachment of the heel from the ground is prohibited. The calculation of maximum forward lean range with respect to the mechanical (anatomical) stability boundary should allow to demonstrate the real safety margin. It is crucial for appropriate assessment of fear and risk of falling.

Clinical assessment of risk of falling is based on Berg Balance Scale (BBS), Tinetti Test or Time Up and Go (TUG) test. These tests aim to evaluate the integration results of the systems involved in postural stability. Unfortunately, clinical tests do not facilitate the understanding the aetiology of the falls-related concerns (13). We postulate more specific and reliable tools to be used for diagnosis and comprehension of the complex postural control processes (14). Since falls usually occur during movements where the COG is shifted near or outside BOS, it seems that the LOS test, is the most appropriate measurement.

The aim of this study was to determine predictive measures of fall in ES and patients with neurodegenerative disorders. For this purpose the clinical and laboratory tests were analysed. We hypothesized that identification of the real functional limit of support with the LOS test allows to assess not only the fear but also the risk of falls. The neurological disorders lead to changes in a mechanism of postural control (4). We assumed that PD patients present different balance strategy when their COG is located near to the stability boundary. The analysis of the sample entropy should allow to identify these changes.

# Methods

Subjects

Sixteen PD subjects and sixteen age-matched healthy controls voluntarily participated in the study. Participants were males and females. A preliminary intragroup comparison with respect to the analysed variables revealed that sex did not differentiate the groups. Based on a medical interview, the general inclusion criteria for PD patients were: age ≥ 65 years, identified and diagnosed Parkinson’s Disease, stage III according to the Hoehn and Yahr Scale. The exclusion criteria were: no consent to participate in the research, dementia or cognitive impairment, neuromuscular, vestibular or orthopedic disorders. PD patients were tested during

„ON period” at their usual antiparkinsonian medication (one hour 1 h after taking their usual

dose of medication). The research was accepted by the Institutional Bioethics Committee.

Table 1. Characteristics of subjects

| Elderly subjects | | | Parkinson’s disease patients | p value | |
| --- | --- | --- | --- | --- | --- |
|  | Sample size | 16 | 16 | - |  |
|  | Age [years] | 67 ± 3 | 63,8 ± 10,5 | 0,187 |  |
|  | Height [cm] | 163,8 ± 5,5 | 167 ± 8,4 | 0,061 |  |
|  | Weight [kg] | 74,8 ± 12,8 | 77,9 ± 11,8 | 0,487 |  |
|  | FES-I (score) | 11,4 ± 5,5 | 12,3 ± 5,4 | 0,984 |  |
|  | Low concern | 5 | 7 | - |  |
|  | Moderate concern | 7 | 3 | - |  |
|  | High concern | 4 | 6 | - |  |
|  | Fellers | 3 | 6 | - |  |
|  | BBS * | 56 (51-56) | 52 (15-56) | 0,034 |  |
|  | FR * | 27 (18-37) | 25 (5-36) | 0,374 |  |
|  | Tinetti test * | 27,5 (27-28) | 27 (8-28) | 0,201 |  |

TUG *	7,24 (5,18-9,11)	9,04 (5,54-21)	0,038

Abbreviations: * median (minimum and maximum) values, FES-I, Falls Efficacy Scale

International; BBS, Berg Balance Scale; FR, Functional Reach; TUG, Time Up and Go.

Apparatus and procedures

The functional stability was evaluated with a force platform (AMTI, Accugait, Watertown, MA, USA). The anthropometrics of the foot were measured with a spreading calliper. The Falls Efficacy Scale International (FES-I) was used to evaluate fear of falling. It includes 7 activities with four possible answers: not all concerned (one point), somewhat concerned (two points), fairly concerned (three points) and very concerned (four points). The highest score (very concerned about falling) is therefore 28 and the minimum (no concern about falling) is 7. Additionally, subjects were asked about their history of the falls which was defined as “*unintentionally coming to rest on the ground or a lower level”* during the preceding year

(15)*.* Information about cause, direction and possible injury from falls were also collected. The LOS test was used to evaluate functional stability and was conducted as described in the study by Juras et al. (2008) (16). This test consists of the three distinct phases. The 1st phase includes quiet standing (QS) and last 10 seconds. During 2nd phase, after an auditory signal, subjects lean forward as quickly and far as they can. The 3rd phase is associated with maintaining the inclined position. The fixation point is placed 3 m away from the participants on the wall in front of them at the eye level. The measurement was interrupted and repeated if subjects lifted their heels. The LOS test lasted 30 seconds and was repeated three times.

The	testing	procedures	involved	also	clinical	tests	widely	applied	for

balanceassessmentt in the ES and PD patients, such BBS, Tinetii test, FR test and TUG test. In three first tests, a higher score indicates a better balance ability. A shorter time in the TUG test suggests a good result.

Data processing

Raw platform data were processed offline using MATLAB (Mathworks Inc., Natick,

MA, USA, v. r2017b). A low-pass 4-order Butterworth filter with a sampling frequency of 7 Hz was used for forces (Fx, Fy, Fz) and moments (Mx, My, Mz), which were later used to calculate the center of foot pressure (COP). Further COP analysis was based on the anatomical foot characteristic. The platform sampling frequency was 100 Hz. The following variables of

COP displacement were calculated for the 1st and 3rd phases of the LOS test: sample entropy

(SempEn) in an anterior-posterior (AP) and medio-lateral (ML) plane (17). In the 2nd phase of LOS test: the range of maximum forward lean was normalized to length from the ankle joint to the head of first metatarsal bone and it was named as a functional forward stability indicator (FFSI). The calculation of the forward functional stability indicator [%] (FFSI) was conducted as follows:

*Figure 1. Functional forward stability indicator*

*Abbreviations NMVE – normalized maximal voluntary COP excursion range calculated from the position of the medial malleolus to the average COP in the third phase of the LOS test corresponding to the maintenance in the inclined position [cm],*

*FFL – the length of the forefoot [cm].*

Statistic

The basic parameters of the descriptive statistics were calculated and analysed. The ShapiroWilk test was used to check the data for normal distribution. The independent-samples t-test was conducted to compare the data from ES and PD patients. The effect sizes for main and interaction effects are reported as d-Cohen. The Spearman rank test was used to evaluate the association between FFSI and FES-I, clinical tests, number and falls. The levels of correlation were considered weak positive (0 – 0,3), weak negative (-0,3 – 0), moderate positive (0,3 – 0,7), moderate negative (-0,3 – (-0,7)), high positive (0,7 – 1,0), high negative (-0,7 – (-1,0))

(18). The level of significance was set at p ≤. 0,05. All calculations were carried out using

STATISTICA v.13.1 (StatSoft, Inc., USA).

# Results

## The analysis of limit of stability COP characteristics between the groups

There were no significant differences between ES and PD patients in FFSI (t= (-0,44); p>0,05; d=0,16). However, it could be observed that ES leaned farther than PD. Also during the 1st phase of the LOS test there were no significant differences in value of SampEn between two groups (t= (-1,99); p>0,05; d=0,73). Significantly lower values of SampEn in the 3rd phase of the LOS test were observed in PD compared to the ES (t= (-2,40); p>0,05; d=0,87).

*Figure 2. The median value of sample entropy in anterior-posterior direction (minimum and maximum marked as a error bars) in the first and third phase of limit of stability test.*

*Abbreviations:: ES, elderly subjects; PD, Parkinson’s disease patients*

## Correlation between FFSI and fear and risk of falling in elderly subjects

A significant positive correlation was observed between FFSI and clinical test such as BBS, FR test and Tinnetti (p< 0,05). There was also a significant negative correlation between FFSI and TUG test, FES-I (p< 0,05). The numbers the falls did not show any correlations with maximum forward lean assessed by FFSI (p> 0,05) (tab. 2).

Table 2. Spearman’s correlation coefficient between FFSI and balance clinical test in ES and

PD

|  | Group | BBS | FR | Tinettii | TUG | FES-I | NoF |
| --- | --- | --- | --- | --- | --- | --- | --- |
| FFSI [%] | ES | 0,634 | 0,674 | 0,759 | -0,874 | -0,831 | -0,229 |
| PD 0,352 0,471 0,698 -0,626 -0,480 -0,604 | | | | | | | |

Abbreviations: ES, elderly subjects; PD, Parkinson’s disease patients; FFSI, Functional forward stability indicator; FES-I, Falls Efficacy Scale International; BBS, Berg Balance Scale; FR, Functional Reach; TUG, Time Up and Go; NoF, number of falls.

## Correlation between FFSI and fear and risk of falling in Parkinson’s Disease patients

There was a significant positive correlation between FFSI and Tinetti test (p< 0,05). Also a significant negative correlation was observed between FFSI and TUG test and number of the falls (p<0,05). BBS, FR test and FES-I did not show any correlations with maximum forward lean assessed by FFSI (p> 0,05) (tab. 2).

Correlation between clinical tests and fear and risk of falling in elderly subjects and PD patients There was only one significant negative correlation between fear of falling and TUG test in a group of ES (p< 0,05). The rest of the clinical test did not correlate with FES-I and a number of the falls Oppositely, in PD patients all clinical tests significantly negatively correlated with FES-I (p< 0,05). The Tinetti test showed significant negative correlations with a number of the falls (p< 0,05). The TUG test significantly positively correlated with the number of the falls (p< 0,05). There were no correlations between BBS, FR test and number the falls (p> 0,05).

Table 3. Spearman’s correlation coefficient between balance clinical tests and FES-I, number

and direction of falls in elderly and PD

| Group | Balance clinical tests | FES-I | Number of falls |
| --- | --- | --- | --- |
| ES | BBS  FR | -0,481  -0,468 | -0,203  -0,313 |
|  | Tinettii | -0,421 | -0,199 |
|  | TUG | 0,703 | 0,134 |

PD	BBS	-0,698	-0,404

| FR | -0,554 | -0,346 |
| --- | --- | --- |
| Tinettii | -0,858 | -0,689 |
| TUG | 0,723 | 0,582 |

Abbreviations: ES, elderly subjects; PD, Parkinson’s disease patients; FES-I, Falls Efficacy Scale International; BBS, Berg Balance Scale; FR, Functional Reach; TUG, Time Up and Go.

# Discussion

FR test and the LOS test are the most frequent measures of functional balance assessing the risk of falls in the elderly population. However, Clark et al. (2005) (19) suggested that differences in task constraints, make FR and LOS tests complementary. Therefore, in this study subjects performed both FR and LOS test. Nevertheless, neither of them differentiated PD patients from their healthy peers. Similar results were reported by Ryckewaert et al. (2015) and Mancini et al. (2008) (20) who noticed no significant differences in forward COP displacement during FR test between PD patients in “ON period” and their control group

Significant differences between PD subjects and their healthy peers were presented by Behrman at al. (2002) (21). They observed significantly lower range of FR distance in PD patients compared to the control groups. According to Behrman at a. (2002) result of FR test below 25 cm indicates a high risk of fall. Although, in our study there were no statistical differences between PD subjects and healthy peers, based on Behrman et al. (2002) result, the group of PD with their result of 22,8 cm of functional distance can be qualified as the risk group.

It is difficult to find norms for the LOS test. Moreover, in the present study, it did not show differences between study groups, what might suggest to be less valuable than FR test. However, it is postulated that the range of maximum forward lean calculated with respect to the anatomical stability boundary allows to demonstrate the real deficit of functional stability

(22). ES used 82,78% of functional BOS which is located between the head of first metatarsal bone and medial malleoulus (ankle joint). Their safety margin equalled 17,22% with respect to

19% of PD subjects. They used smaller area of the functional BOS during the LOS test (fig. 3). Existence of safety margin indicates deficits in balance. The greater safety margin is combined with greater postural instability (10). It is obvious, that appropriate rehabilitation improves balance (23). Therefore, purposeful therapy should be aimed to reduce safety margin and allow to relocate COP near the anatomical stability boundary during LOS test. FFSI bears clear information about functional stability deficits in the sagittal plane, and it also indicates a target value in the range of maximum forward COP excursion for patients in balance rehabilitation.

*Figure 3. Forward stability region*

*Abbreviations:: ES, elderly subjects; PD, Parkinson’s disease patients; FFSI, Functional forward stability indicator; FASL, Forward anatomical stability limit; MS margin safety*

Computerized posturography should be considered a more appropriate measure of balance ability among healthy ES than subjective tests. In the present study FFSI highly correlated with FOF in the control group. The decreased range of maximum forward lean indicated an increased postural threat. In turn, there were no correlations between FR test and both the FOF and history of falls in healthy ES. LOS test is more specific than FR test because it stresses the use of the ankle strategy (24). Thanks to this strategy the center of mass is repositioned by moving the whole body as a single-segment inverted pendulum by production of torque at the ankle (25). This minimizes early and small perturbation of upright posture which is crucial with respect to efficient postural control. Furthermore, other studies have shown subjects exhibiting the same reaching distance in FR which adopt different movement strategies during the test. These strategies may significantly affect the final result and includes rotation of the trunk and combination of the ankle and hip strategy (26). Therefore, the LOS test being less prone to such strategy alterations during the performance, is in our opinion, more appropriate to early identification of balance deficits.

Other clinical balance test as well as FR test are poor predictors of fear of falling in groups of healthy ES. In the present study only the TUG test correlated with the FES-I. Neither of them correlated with a history of falls. Landers et al. 2015 (27) showed which self-reported questionnaires are most clinically applicable to the prediction of falls in the elderly population. They also indicated that the TUG test is the most predictive clinical test of risk of falls. In general, clinical tests detect visible balance deficits. However, an identification of postural control changes that are invisible to a naked eye should be pursued. FFSI allows not only to detect the area of the safety margin, but also it is correlated with FOF in healthy ES. Moreover, FFSI also highly correlated with clinical test such a TUG, Tinneti test, which are known to be good predictors of risk of falls (28). We can conclude that based our results, it is not necessary to use the whole battery of clinical tests for balance assessment and still achieve satisfactory diagnostic outcomes. Acquiring more information in less time is especially important when conducting diagnosis in specific populations and situations.

Diagnosis of the PD subjects led to different observations. According to the Hoehn and Yahr Scale all PD subjects in this study were in III state. Everyone had a visible and persistent changes in postural control (2), which could be detected by both clinical and posturography measurements. All clinical test highly correlated with FOF. Additionally, Tinetti and TUG test highly correlated with a history of falls. Nevertheless, FFSI had a poor correlation with FOF, but it is highly correlated with a history of falls. Moreover FFSI was associated only with two clinical tests of risk of falls. Therefore, we postulate that the balance assessment of the PD population should be made both with the clinical test and the computerized posturography.

As it occurred the differences between PD subjects and healthy peers can be easily detected with BBS and TUG test. Although the TUG test is an incontestably appropriate measure to assess the dynamic balance among PD subjects, using BBS for dynamic balance assessment is more questionable (29). Some authors indicated that the BBS, when used to measure balance in patients with PD, may not completely assess the whole spectrum of postural control impairments typical for this disease (30). Moreover La Porta et al. (2015) (31) suggested that the BBS, even if modified by the new rating scale, may not be an effective tool for the measurement of early postural control disturbance in patients with PD.

Although there were no differences between PD subjects and the control group, it could be observed that the LOS test was more demanding for PD subjects. During 3rd phase of the test where subjects had to maintain inclined position, PD subjects were characterized by lower values of Sample entropy. According to Richmanand and Moorman (2000) low values of entropy show more regularity in the COP signal. Thereby, less chaotic excursions may be defined as a characteristic of an unsuccessful vigilant strategy to keep balance. Furthermore, Donker et al. (2008) (32) showed that the amount of attention invested in keepingan upright posturee is positively correlated with sway regularity. Based on these report PD subjects paid more attention to keeping inclined position than the control group (fig. 2). Reinert et al. (2017) (33) confirmed that the use of nonlinear analysis methods such as sample entropy may improve the ability of the LOS test to identify differences in performance that are not currently revealed by traditional sway ranges.

This study has several limitations. A larger sample size would have been better as it would help to better generalize the results. Additionally, it is known that psychological factors, which had not been taken into account in this study, are associated with the increased postural threat also could affect the processing of postural control. Finally, it is known that pharmacological treatments may have an impact on postural control in PD patients. Although we did our best to satisfy the standardization of procedures we did not explore the problem of the impact of medication on postural control.

# Conclusion

The present study highlights the importance of a quantitative approach for balance evaluation in ES and also elderly with a neurological disorders. Functional forward stability indicator is a good predictor of fear of falling in group of elderly people. Additionally, it allows to show real balance deficits both in PD patients and healthy peers without using external reference group or created norms. A complete assessment of postural balance in PD patients should be made both with posturography measurement and clinical test.

# Acknowledgements

The study was supported by the National Center for Research and Development Grant under

the	program	STRATEG	MED	III	within	the	“VB-Clinic”	project	no.

STRATEGMED3/306011/1/NCBR/2017.

**Conflict of interest statement**

All authors confirm there are no known conflicts of interest

# Bibliography

1. Haagsma JA, Olij BF, Majdan M, Beeck EF Van, Vos T, Castle CD, et al. Falls in older aged adults in 22 European countries : incidence , mortality and burden of disease from 1990 to 2017. Inj Prev. 2020;1–8.
2. Lee HK, Altmann LJP, McFarland N, Hass CJ. The relationship between balance confidence and control in individuals with Parkinson’s disease. Park Relat Disord.

2016;26:24–8.

3. Tangen GG, Bergland A, Engedal K, Mengshoel AM. The importance of parkinsonian signs for gait and balance in patients with Alzheimer’s disease of mild degree. Gait Posture . 2017;51:159–61.
4. Stolze H, Klebe S, Zechlin C, Baecker C, Friege L, Deuschl G. Falls in frequent neurological diseases: Prevalence, risk factors and aetiology. J Neurol. 2004;251(1):79–84.
5. Park JH, Cho H, Shin JH, Kim T, Park SB, Choi BY, et al. Relationship among fear of falling, physical performance, and physical characteristics of the rural elderly. Am J Phys Med Rehabil. 2014;93(5):379–86.
6. Del-Río-Valeiras M, Gayoso-Diz P, Santos-Pérez S, Rossi-Izquierdo M, FaraldoGarcía A, Vaamonde-Sánchez-Andrade I, et al. Is there a relationship between short FES-I test scores and objective assessment of balance in the older people with ageinduced instability? Arch Gerontol Geriatr. 2016;62:90–6.
7. Kempen GIJM, Todd CJ, Van Haastregt JCM, Zijlstra GAR, Beyer N, Freiberger E, et al. Cross-cultural validation of the Falls Efficacy Scale International (FES-I) in older people: Results from Germany, the Netherlands and the UK were satisfactory. Disabil Rehabil. 2007;29(2):155–62.
8. Maki BE, McIlroy WE. Control of rapid limb movements for balance recovery: Agerelated changes and implications for fall prevention. Age Ageing.

2006;35(SUPPL.2):12–8.

9. Forth KE, Fiedler MJ, Paloski WH. Gait & Posture Estimating functional stability boundaries for bipedal stance. Gait Posture . 2011;33(4):715–7.
10. Błaszczyk J.W., Lowe D. L. HPD. Ranges of postural stability and their changes in the elderly. Gait Posture. 1994;2:11–7.
11. Chan CW, Rudnis A. Foot Biomechanics During Walking and Running. Mayo Clin

Proc. 1994;69(5):448–61.

12. Duarte M, Zatsiorsky VM. Effects of body lean and visual information on the equilibrium main- tenance during stance. Exp Brain Res. 2002;60–9.
13. Pauelsen M, Vikman I, Johansson Strandkvist V, Larsson A, Röijezon U. Decline in sensorimotor systems explains reduced falls self-efficacy. J Electromyogr Kinesiol.

2018;42(May):104–10.

14. Kamieniarz A, Michalska J, Brachman A, Pawłowski M, Słomka KJ, Juras G. A posturographic procedure assessing balance disorders in parkinson’s disease: A

systematic review. Clin Interv Aging. 2018;13:2301–16.

15. Jean-Baptiste Mignardot, Olivier Beauchet CACC, Deschamps T. Postural sway, falls, and cognitive status: a cross-sectional study among older adults. J Alzheimers Dis.

2014;41(2):431–9.

16. Juras G, Słomka K, Fredyk A, Sobota G, Bacik B. Evaluation of the Limits of Stability (LOS) Balance Test. J Hum Kinet. 2008;19(1):39–52.
17. Richman JS, Moorman JR. Physiological time-series analysis using approximate entropy and sample entropy. AmJ Physiol Hear CircPhysiol. 2000;278:H2039–49.
18. Ratner B. The correlation coefficient: Its values range between 1/1, or do they. J

Targeting, Meas Anal Mark. 2009;17(2):139–42.

19. Clark S, Iltis PW, Anthony CJ, Toews A. Comparison of Older Adult Performance

During the Functional-Reach and Limits-of-Stability Tests. J Aging Phys Act. 2005;13:266–75.

20. Mancini M, Rocchi L, Horak FB, Chiari L. Effects of Parkinson’s disease and levodopa on functional limits of stability. Clin Biomech. 2008 May;23(4):450–8.
21. Behrman AL, Light KE, Flynn SM, Thigpen MT. Is the functional reach test useful for identifying falls risk among individuals with Parkinson’s disease? Arch Phys Med Rehabil. 2002;83(4):538–42.
22. Słomka KJ, Michalska J, Marszałek W, Bacik B, Juras G. Forward functional stability indicator (FFSI) as a reliable measure of limits of stability. MethodsX. 2020;7:10–6. 23.	Lesinski M, Hortobagyi T, Muehlbauer T, Gollhofer A, Granacher U. Effects of Balance Training on Balance Performance in Healthy Older Adults: A Systematic Review and Meta-analysis. Sport Med. 2015;45(12):1721–38.
24. Horak FB. Postural orientation and equilibrium: What do we need to know about neural control of balance to prevent falls? Age Ageing. 2006;35(SUPPL.2):7–11.
25. Horak F, Kuo A. Postural adaptation for altered environments, tasks, and intentions. In:

Biomechanics and neural control of posture and movement. Springer; 2000. p. 267–81.

26. Kage H, Okuda M, Nakamura I, Kunitsugu I, Sugiyama S, Hobara T. Measuring Methods for Functional Reach Test: Comparison of 1-Arm Reach and 2-Arm Reach.

Arch Phys Med Rehabil. 2009;90(12):2103–7.

27. Landers MR, Oscar S, Sasaoka J, Vaughn K. Balance Confidence and Fear of Falling Avoidance Behavior Are Most Predictive of Falling in Older Adults: Prospective Analysis. Phys Ther. 2015;96(4):433–42.
28. Ambrose AF, Paul G, Hausdorff JM. Risk factors for falls among older adults: A review of the literature. Maturitas. 2013;75(1):51–61. Available from:
29. Boulgarides LK, Mcginty SM, Willett JA, Barnes CW. Use of Clinical and

Impairment-Based Tests to Predict Falls by Community-Dwelling Older Adults. Phys Ther. 2003;83(4):328–39.

30. King LA, Priest KC, Salarian A, Pierce D, Horak FB. Comparing the Mini-BESTest with the Berg Balance Scale to evaluate balance disorders in Parkinson’s disease.

Parkinsons Dis. 2012;2012.

31. La Porta F, Giordano A, Caselli S, Foti C FF. Is the Berg Balance Scale an effective tool impairments in patients with Parkinson’s disease? for the measurement of early postural control Evidence from Rasch analysis. Eur J Phys Rehabil Med. 2015;51:705–

16.

32. Donker SF, Ledebt A, Roerdink M, Savelsbergh GJP, Beek PJ. Children with cerebral palsy exhibit greater and more regular postural sway than typically developing children. Exp Brain Res. 2008;184(3):363–70.
33. Reinert SS, Kinney AL, Jackson K, Diestelkamp W, Bigelow K. Age stratification and sample entropy analysis enhance the limits of stability tests for older adults. J Appl

404	Biomech. 2017;33(6):419–23.

405

[Click here to download Figure figure 1.tif](https://www.editorialmanager.com/pone/download.aspx?id=26294903&guid=bc1f86c4-2f7d-4394-b929-f7053f3e20b1&scheme=1)

[Click here to download Figure figure 2.tif](https://www.editorialmanager.com/pone/download.aspx?id=26294904&guid=cca1fbb2-2e18-4692-9d41-dd99f8b1db13&scheme=1)

[Click here to download Figure Figure 3.tif](https://www.editorialmanager.com/pone/download.aspx?id=26295018&guid=aeb2f7e8-b28d-4124-88dc-abcbeb38b3b2&scheme=1)

Figure 3_300.jpg

data sheet

[Click here to access/download](https://www.editorialmanager.com/pone/download.aspx?id=26295497&guid=a3ca9a4c-45ce-4389-a6a3-654ff2633d6c&scheme=1)

[Supporting Information data sheet.csv](https://www.editorialmanager.com/pone/download.aspx?id=26295497&guid=a3ca9a4c-45ce-4389-a6a3-654ff2633d6c&scheme=1)
