# Supplementary material for: Fall-related measures in elderly individuals and Parkinson’s disease subjects
Source: PLoS One. 2020 Aug 13;15(8):e0236886. doi: 10.1371/journal.pone.0236886 (PMC7425912; doi:10.1371/journal.pone.0236886)
Supplement: Attachment — Submitted filename: PONE-D-20-08972_R1_reviewer...response comments.docx [file pone.0236886.s004.docx]

PLOS ONE

Fall-related measures in elderly individuals and Parkinson’s disease subjects

--Manuscript Draft--

| Manuscript Number: | PONE-D-20-08972R1 |
| --- | --- |
| Article Type: | Research Article |
| Full Title: | Fall-related measures in elderly individuals and Parkinson’s disease subjects |
| Short Title: | Falls in elderly and Parkinson's Disease |
| Corresponding Author: | Justyna Michalska, PhD  The Jerzy Kukuczka Acadamy of Physical Education Katowice, POLAND |
| Keywords: | limit of stability test, fear of falling, safety margin, postural control, balance clinical test |
| Abstract: | Falls pose a serious problem in elderly and clinical populations. Most often, they lead to a loss of mobility and independence. They might also be an indirect cause of death. The aim of this study was to determine an objective predictor of the fear of falling and falls in elderly subjects (ESs) and Parkinson’s disease (PD) subjects. Thirty-two ESs were examined in this study, of whom sixteen were diagnosed with PD. The testing procedures comprised force plate measurements (limit of stability test–LOS test) and clinical tests (Berg Balance Scale, Functional Reach Test, Timed Up and Go test, Tinetti test). The Falls Efficacy Scale International (FES-I) was used to evaluate the fear of falling. The range of the maximum forward lean was normalized to the length from the ankle joint to the head of the first metatarsal bone and was named the functional forward stability indicator (FFSI). The FFSI, derived from the LOS test, allowed us to demonstrate the real deficit in functional stability and individual safety margins. Moreover, the FFSI was highly correlated with the FES-I score and all clinical test results in elderly subjects (r>0,6; p<0.05). In PD subjects, the FFSI was poorly correlated with the fear of falling, the Berg Balance Scale score and the Functional Reach distance; however, a high correlation with the number of falls (r>0,6, p<0.05) was noted. The PD subjects presented a different balance strategy when close to their stability limits, which was also reflected in the lower values of sample entropy (t=(2.40); p>0.05; d=0.87). The FFSI might be a good predictor of the fear of falling in the group of elderly people. Additionally, the FFSI allows us to show real balance deficits both in PD subjects and in their healthy peers without the need for a reference group and norms. In conclusion, it is postulated that the popular clinical assessments of postural balance in PD subjects should be accompanied by reliable posturography measurements. |
| Order of Authors: | Justyna Michalska, PhD |
|  | Anna Kamieniarz |
|  | Anna Brachman |
|  | Wojciech Marszałek |
|  | Joanna Cholewa |
|  | Grzegorz Juras |
|  | Kajetan J. Słomka |
| Response to Reviewers: | Editor comment:   1. Please ensure that your manuscript meets PLOS ONE's style requirements,including those for file naming.   Answer:  In the current revision we have followed all the PLOS ONE style requirements including those for file naming.   2. Please provide additional details regarding participant consent. In the ethics |

|  | statement in the Methods and online submission information, please ensure that you have specified (1) whether consent was informed and (2) what type you obtained (for instance, written or verbal, and if verbal, how it was documented and witnessed). If your study included minors, state whether you obtained consent from parents or guardians. If the need for consent was waived by the ethics committee, please include this information.  Answer:  The additional details regarding participant consent were provided. The following sentences were added to the methods section: “The subjects gave informed written consent for voluntary participation in the study. The research was approved by the Institutional Ethics Committee of the Medical University of Warsaw (number  KB/28/2014).”   3. Please ensure that you refer to Figure 1 in your text as, if accepted, production willneed this reference to link the reader to the figure.   We have reformatted the equation in Word’s “Equation Tools” and removed equation as a figure form current manuscript. It is now referred to as an equation.   4. We note you have included a table to which you do not refer in the text of yourmanuscript. Please ensure that you refer to Table 1 in your text; if accepted, production will need this reference to link the reader to the Table.   Answer:  The reference in the text to Table 1 was added: …“Sixteen PD subjects and sixteen anthropometrically matched healthy controls voluntarily participated in the study (Table  1).”   5. Please include captions for your Supporting Information files at the end of yourmanuscript, and update any in-text citations to match accordingly.   We have added Supporting Information files at the end of the manuscript.    Reviewer #1  Dear authors, Thank you for your submission and your contribution to the field. This study is interesting with some worthy outcomes. I have attached a copy of your submission with some comments and suggestions. In summary, my main reason for recommending a major review is the lack of focus in the paper and also some paragraphs are difficult to understand. I suggest to review what precise questions you are asking of the data, clearly formulate hypotheses based on previous research and stick with these tightly throughout the paper. Points are made in the introduction but especially the discussion that are not directly related to the questions asked in this paper and can be shortened or removed. Overall it is a good paper and after some changes will be ready for publication.  Answer:  First of all, we would like to thank the reviewer for a very insightful review. The manuscript was amended thoroughly according to the reviewer’s comments and suggestions. Some issues have risen after reading the review, which is explained in detail below. Referring to the reviewer's concerns about language, we have sent our manuscript to The American Journal Experts (the certificate was attached). So all the proofread suggestions have been done.  Reviewer comment  The paragraph (lines 55-59/after correction lines 55-59) is not very clear to me..I understand that increased fear of falling may be a reaction to physical/cognitive deteriorations that can lead to beneficial cautious behaviour but it can also exacerbate the risk of falling.  Answer:  To clarify this sentence we have provided changes which are the following:  Lines 51-56: “The fear of falling (FOF) could appear well before and/or after an incident of fall which creates a vicious cycle. Concomitant psychological symptom of falls is the fear of falling, which is common among older adults whether or not they have sustained a fall. FOF might also be a proper reaction to certain situations, leading the elderly to be cautious behaviour, and can contribute to fall prevention through careful choice of movement activity. Nevertheless, ESs increasingly cease physical activity because of the FOF (5).” |
| --- | --- |

|  | Reviewer comment  Lines 80-86: This problem statement needs to be made clearer .I believe you wish to compliment the disadvantages of clinical measures with more objective measures of stability however this is not directly stated.  Answer:  We have added following sentence to clarify the problem:” Clinical tests are often based on a qualitative assessment (BBS, Tinetti test). Despite the clear assessment instructions, the researcher may influence the test results.”  Reviewer comment  Your aim is not specifically addressing the problem statement in the previous paragraph… too general…perhaps with the aim of finding objective predictors of fall risk that can support/replace clinical assessments while also being specific to the patient needs is what you are aiming at? ..sample entro-py is also not described.  Answer:  We agree with the reviewer, that the aim of our study is too general, so we have changed this paragraph as follows:  Lines: 101- 111 - “The aim of this study was to determine an objective predictor of the fear of falling and falls in ESs and patients with neurodegenerative disorders. For this purpose, the clinical and laboratory tests were analyzed. Depending on the study groups, the proposed objective measure could replace or support clinical assessment. Posturographic examination can also register early and nonvisible postural control changes, so we assumed that this objective assesment might replace clinical tests among ESs. We also hypothesized that the identification of the real functional limit of support with the LOS test would allow us to assess not only the fear of falling but also the risk of falls. Neurological disorders lead to changes in the mechanism of postural control (4). We assumed that PD patients present different balance strategies when their COG is located near their stability boundary. The analysis of the sample entropy should allow us to identify these changes.”  We believe that these corrections would facilitate reading and understanding the discussion.  Reviewer comment  Include number of males and females in each group.  Answer:  This information has been added in table 1. There were 9 males and 7 females in the PD group, and 8 males and 8 females in control group.  Reviewer comment  How did you ensure there was no significant cognitive impairment in the PD sample?  Answer:  PD subjects had the results of the Mini Mental State Examination (MMSE) in their medical history. All of them obtained more than 26 points. We have added this information in eligibility criteria as follows: “… no consent to participate in the research; dementia or cognitive impairment (MMSE score below 26 points); and neuromuscular, vestibular or orthopedic disorders “  Reviewer comment  Summary statistics for all measurements should be presented, whether they are significant or not…not just sample entropy.  Answer:  All results have been presented in the manuscript – significant or not. The reviewer might have not noticed this fact because in order not to duplicate the data presentation the results of the comparison between groups are located in the main text of the manuscript (FFSI, sampEn) and in table 1 (FES-I, BBS, FR, Tinetti test, TUG). We described the results of FFSI and SampEn in details, because we built our conclusion based on these observations. All correlations are presented in table 2 and 3. |
| --- | --- |

|  | Reviewer comment  A summary of the findings in your study and if they met or did not meet your hypotheses would be good to start with.  Answer:  Thank you for this comment. Following this suggestion we have pointed our hypothesis in our discussion as follows:  1)Line 256-257: “…So the existence of a safety margin might indicate deficits in balance, thus our research hypothesis was validated…”  2)Line 292-295: “…We can conclude that, based on our results, it is not necessary to use the whole battery of clinical tests for balance assessment to achieve satisfactory  diagnostic outcomes, and our research hypothesis was validated.…”  3)Line 304-306 “…Our results validated our research hypothesis; therefore we postulate that balance assessments of the PD population should be made both with clinical tests and with computerized posturography …”  4)Line 326-327: “…Therefore, we confirmed our research hypothesis that the LOS test is a more challenging motor task for PD patients …”  Reviewer comment  This paragraph (221-233/after correction 252-266) needs to be made clearer… there are many points being made that are not aligned, making it difficult to read.  Answer:  Thank you for pointing this out. We have revised this paragraph and made all necessary grammar and spelling corrections. We believe that it is now more clear. We have also edited and added following sentences: “A greater safety margin decreases the range of the maximum forward lean, thereby decreasing functional movement. So the existence of a safety margin might indicate deficits in balance…”  Reviewer comment  Figure 3 should be in the methods in my opinion  Answer:  We appreciate this comment, however figure 3 (after correction figure 2) already presents some of results , therefore we put it in the discussion section.  Reviewer comment  Line: 239-252(after corrections lines:272-282) Much of this can be summarized in a smaller number of sentences  Thank you for this comment. The paragraph was revised and made more concise for the sake of clarity and readability. The number of sentences was reduced.  Reviewer comment  Line: 265-267 (after corrections lines 279-282)“Therefore, the LOS test being less prone to such strategy alterations during the performance, is in our opinion, more appropriate to early identification of balance deficits” – evidenced or opinion?  Answer:  We fully understand reviewer’s concern. We have changed our hypothesis as follow: Depending on the study groups, the proposed objective measure could replace or support clinical assessment. Posturographic examination can also register early and nonvisible postural control changes, so we assumed that this objective assessment might replace clinical tests among ESs.” Therefore, we have presented our opinion based on the results.  Reviewer comment  Line 277-280 (after correction lines 292-296) “We can conclude that based our results, it is not necessary to use the whole battery of clinical tests for balance assessment and still achieve satisfactory diagnostic outcomes. Acquiring more information in less time is especially important when conducting diagnosis in specific populations and situations.” – was this an aim? |
| --- | --- |

|  | Answer:  Once again we fully understand reviewer’s concern. We have changed our hypothesis, so statement in the current form correspond to the aim of our study.  Reviewer #2 Introduction  Reviewer comment 1:  General – language. For publication in PLOS ONE, the article should be “presented in an intelligible fashion and is written in standard English”. The authors need to spell check and go through their manuscript. Getting language editing help is recommended.  Answer:  At the beginning, we would like to express thanks to the reviewer for a very insightful review. The manuscript was corrected according to the reviewer’s suggestions. Your comments have contributed to improvement our paper. The manuscript was proofread by a certified proofread agency (certificate attached in the files).  Reviewer comment 2:  Why is there no citation after “The ES experience falls due to significant deterioration of balance ability associated with decrease of muscle strength, sensory function and widespread degeneration in the central nervous system.”?  Answer:  Thank you for pointing this out. We have added missing citation: Ambrose AF, Paul G, Hausdorff JM. Risk factors for falls among older adults: A review of the literature. Maturitas.2013; 75 (1): 51-61.  Reviewer comment 3  Please revise the unclear sentence “The questionnaire refers to the most of the basic, everyday activities presented by disabled people would be likely to have difficulty with because of their FOF. :  Answer:  We have revised this sentence:“The questionnaire refers to the most of the basic, everyday activities. Only people with FOF may have difficulty to perform these actions.”  Reviewer comment 4  Limit of Stability is not abbreviated at the first time of use.  Answer:  Thank you for pointing this out. We have abbreviated Limit of Stability (LOS) at the first time of use .  Reviewer comment 5  The calculation of maximum forward lean range with respect to the mechanical  (anatomical) stability boundary should allow to demonstrate the real safety margin. It is crucial for appropriate assessment of fear and risk of falling.” – Says who? Says you? Nothing in this sentence supports just using forward lean range. Medio-lateral leaning and LOS could be meaningful as well! Please revise and use references when building argumentation. Also, you can never be sure that you reach the “real” safety margin, as participants may stop themselves from leaning as far as they possibly can out of fear of falling or reduced joint flexibility. It is very difficult to achieve the “real” safety margin. I suggest you rephrase to something like “ should allow us to reach the boundary that is in close proximity to the real safety margin”  Answer:  Thank you for rising this issue. To clarify why we focused our research only on the forward stability boundary, we have added the following sentences to the introduction: “In older adults over 65 yr, 60% of falls occur in a forward direction (O’Neill et al. 1994). Additionally, Youn et al. (2017) noticed that more than 70% of PD patients had a history of falls in the forward direction. Therefore, in the present study, we focused on exploring the anterior stability boundary”. We also agree with the reviewer, that we can never be sure that we reach the “real” safety margin, as participants may stop themselves from leaning as far as they possibly can out of the fear of falling or reduced joint flexibility. Therefore, we have rephrased this sentence according to the reviewer’s suggestion: “The calculation of the maximum forward lean range with respect to the mechanical (anatomical) stability boundary should allow subjects to reach the boundary that is in close proximity to the real safety margin”. We also have added |
| --- | --- |

|  | references: Błaszczyk J.W., Lowe D. Ranges of postural stability and their changes in the elderly. Gait & Posture 1994(2):11-7. The authors have pointed out the importance of the detection of safety margin: “Reduction of the maximum voluntary excursion increases the probability of recovery from postural instability by increasing the margin safety, which allows for more time to complete the recovery programme”.  Reviewer comment 6  “Since falls usually occur during movements where the COG is shifted near or outside BOS, it seems that the LOS test, is the most appropriate measurement.” – Again, no references to support this statement. Also if falls usually occur during movements, is this really associated with a LOS test where a participants stands bilaterally, relatively static, and leans in different directions? Shouldn’t it rather be associated with people walking, stepping and how the COG moves within the LOS then?  Answer:  We agree that this statement should be supported by proper reference. However, this statement was created based on knowledge about postural strategy. Upright posture is controlled during disturbance by the ankle and hip joint movements, classified as the “ankle strategy” and “hip strategy”, respectively. The greater the postural disturbance, the closer the COG moves to the stability boundary. When the COG crosses the stability boundary, the subject has to take a step to recover the upright posture. According to Clark et al. (2005), for many older adults, spatial and temporal control of the COG is decreased, and movement strategies become unwieldy, resulting in an increased risk of falls when performing reaching tasks or gait initiation. Therefore, we have rephrased the sentence as follows: “With an inefficient postural strategy (ankle and hip strategy) in elderly individuals, falls usually occur during movements where the COG is shifted near or outside the BOS. It seems that the LOS test is the most appropriate measurement.” We have also added the appropriate reference (Cleworth et al. 2018 Gait & Posture).  Methods:  Reviewer comment 1  The recruitment and inclusion procedure is not described thoroughly. It says that sixteen PD subjects and sixteen age-matched healthy controls voluntarily participated. Was this a convenience sample? How did recruitment proceed? Was this done at a hospital? Were the controls matched only on age? How will eventual non-matched variables confound your results?  Answer:  Again, thank you for pointing this out. We have provided several corrections in the description of this section. We recruited PD subjects from the Local Association of Parkinson's disease community. We have changed the phrase from “PD patients” on “PD subjects”. One of our co-authors, who is the specialist of PD physiotherapy verified the inclusion and exclusion criteria. Subjects who met eligibility criteria were invited to examination. The research took place at the Academy of Physical Education in Katowice (Poland). The sample size of PD subjects was determined by the number of subjects from the Association of Parkinson's Disease who met the eligibility criteria. The control group was matched not only for age, but also for body height and weight. Table 1 presents the anthropometric values of the two groups. All of the above details have been included in the manuscript.  Reviewer comment 2  Please indicate how many men and women there were  Answer:  Thank you for this comment. This information was added to the manuscript (table 1). In the PD group there were 9 males and 7 females, in control group there were 8 males and 8 females.  Reviewer comment 3  The research was accepted by the Institutional Bioethics Committee.” – Where is this committee situated? More details required.  Answer:  We have changed the sentence as follow: “The research was accepted by the Institutional Ethics Committee Medical University in Warsaw number KB/28/2014”.  Reviewer comment 4 |
| --- | --- |

|  | Table 1 – p-values are given but can be misinformative when there is risk for low statistical power. I encourage the authors to present confidence intervals instead. Also, the statistic section says that normal distribution was controlled for, but there is no information whether the parameters were normally distributed or not. Also, the “independent-samples t-test was conducted”. Did you use a parametric or nonparametric t-test?  Answer:  We agree that the section of statistical methods should be written with more details. Depending on the variable distribution, parametric or nonparametric statistical analysis was used. All anthropometric variables, the FFSI and the SampEn, were normally distributed compared to FES-I, BBS, TUG, FR and Tinetti test scores; therefore, we used parametric and nonparametric tests, respectively. To compare values of age, weight, body weight, the FFSI and the SampEn between groups, an independentsample t-test was conducted. To compare FES-I, BBS, TUG, FR and Tinetti test scores, the Mann-Whitney U test was conducted. All correlations were calculated by the nonparametric Spearman rank test. We have corrected this section as follows: “The parametric and nonparametric tests were used. An independent-sample t-test was conducted to compare the anthropometric variables FFSI and Samp En from ESs and PD patients. The Mann-Whitney U test was conducted to compare FES-I, BBS, Tinetti, FR and TUG test scores from ESs and PD patients.” We have removed p-values from table one and we have added confidence intervals.  Reviewer comment 5  Table 1 – I suggest putting percentages after the values on low-moderate-high concern to make it clearer.  Answer:  We have put percentages after the values on low-moderate-high concern in table 1.  Reviewer comment 6  Table 1 – for BBS, FR, Tinetti test and TUG; indicate what unit is presented (score, seconds, etc.), e.g. “TUG (s)”  Answer:  Table 1 was revised according to reviewers comments.  Reviewer comment 7  From the methods it becomes clear that the study investigates fall history and not prospective falls. This should be indicated in the manuscript title as “history of falls”. This means that the outcome is prevalent before the risk factor is measured, and could likely influence the results. This should be mentioned in the limitation section.  Answer:  Once again, thank you for pointing this out. We have changed the title for: ” Fall-related measures in elderly individuals and Parkinson’s disease subjects”. We also added to the limitation section following sentences: “Future research should be focused on prospective risk of falls. Future research should be focused on the prospective risk of falls. It will be useful to create FFSI norms that can help diagnose the risk of falls.”  Reviewer comment 8  The forward leaning LOS test is used. The advantages and disadvantages of this test needs to be discussed more. What information on LOS is missed? The authors early cite Forth et al. 2011 that in their paper states “However, an individual’s functional stability boundary during bipedal stance is more likely an ellipse much smaller than the base of support” The forward lean LOS test does not cover the other directions in an ellipse, and falls can occur in several directions. If the manuscript should be placed in the context of fall prediction, the validity of the test, e.g. strength and weaknesses should be properly discussed.  Answer:  Thank you for pointing this out, as we have mentioned before, we focused on forward stability boundary, because of the frequency of falls in this direction. We wrote about some advantages and disadvantage of the LOS test in section of discussion. |
| --- | --- |

|  | Reviewer comment 9  “The LOS test lasted 30 seconds and was repeated three times.” – were all three values analyzed? Or did you use the maximal value? Or an average over the three? Please specify. If using an average – discuss if you think test fatigue might influence the results.  Answer:  Thank you for this comment. We have added calculation of the intraclass correlation coefficients for the limit of stability test in the statistical section as follows: “The levels of correlation were considered weak positive (0 – 0,3), weak negative (-0,3 – 0), moderate positive (0,3 – 0,7), moderate negative (-0,3 – (-0,7)), high positive (0,7 – 1,0), and high negative (-0,7 – (-1,0)) (18). Intraclass correlation coefficients (ICCs) were calculated for the LOS test (FFSI, sampEn phase 1, sampEn phase 3). The levels of reliability were considered poor (ICC < 0.50), moderate (0.50 ≤ ICC < 0.75), good (0.75 ≤ ICC < 0.90), and excellent (ICC ≥ 0.90), according to Portney et al (2000). The LOS test achieved excellent reliability with 3 repetitions..” – this of course means that we took average of three trials for further analysis. The fatigue probably might influence the result of the test if there were not rest break between repetitions. That is why each research subjects were getting off the force plate after each trial and could rest.  Reviewer comment 10  I suggest helping the reader understand the FFSI better by adding this parameter its unit of measure (%?) to Table 1  Answer:  Thank you for this suggestion. We have added measurement units of FFSI to the Table 1.  Results  Reviewer comment 1  Table 2 – what does the red numbers indicate? Please specify  Answer:  Again, thank you for the comment. Indeed, the red color might be misleading. So we have changed red to bold indicating statistical significance. The description of this was added to the table’s legend.  Reviewer comment 2  Table 3 – it is said to report “number and direction of falls”. Please specify, did you record in what direction the participants had fallen? And if so, this is not reported in table 3.  Answer:  Thank you for pointing this out. It was a mistake. We didn’t examine the direction of the falls. So we have corrected the title of Table 3 as follows: “Spearman’s correlation coefficients between among balance clinical test scores, the FES-I score, and the number of falls in elderly subjects and PD subjects.  Discussion  Reviewer comment 1  Line 235-236 “Computerized posturography should be considered a more appropriate measure of balance ability among healthy ES than subjective tests.” What about the fact that most objective postural stability measures are performed in a static stance, while clinical tests are more dynamic in their nature? And falls often occur during movement and everyday situations, and not necessarily when standing still – for example, see Robinovitch et al. 2013 in the Lancet that have video-recorded falls.  Answer:  We fully understand the reviewer’s concern. We agree that most objective measures are performed in a static stance, but falls predominantly occur during more dynamic motor tasks. Therefore, we chose the limit of stability test rather than quiet standing. Almost 30 years ago, Duncan et al. (1990) created a simple measure such as the Functional Reach Test, which was highly correlated with the risk of falls. Over the years, it turns out that indications of the functional limit of stability are crucial to assess the risk of falls. Even simple motor tasks such as body leaning on the force plate are |
| --- | --- |

|  | very informative. We know exactly where the COP is located within the base of support. This allows for the calculation of the safety margin. No clinical test allows this. Additionally, having a physiotherapist’s experience, one can notice that the researcher has a large influence on the BBS and Tinetti test results.  Reviewer comment 2  Line: 254-255 “However, an identification of postural control changes that are invisible to a naked eye should be pursued.”  Line 258-260 “We can conclude that based our results, it is not necessary to use the whole battery of clinical tests for balance assessment and still achieve satisfactory diagnostic outcomes.”  These statements require a better rationale. Why should we use objective measures? For fall risk? Can you really be sure this is better? There is evidence that self-reported fall history is a better predictor of prospective falls than objective tests, see for example Johansson et al. 2019 in Human Movement Science. So why should clinics spend resources on expensive equipment when it is better to ask a simple question?  Answer:  We fully understand reviewer’s concern, however self-reported fall history assesses what happened in the past. We agree that, based on this report, research subjects have poor balance. However, is it possible to predict a fall before the first accident? The fear of falling can appear both before and after accidents or falls. In our study, we detected the fear of falling without a history of falls in almost all research subjects. Therefore, our indicator has great potential to determine the fear of falling. There is a high probability of detecting the risk of falling if the research is conducted with follow-up examinations. Thank you for suggesting Johansson et al. papers, we have added it to references  Reviewer comment 3  Line: Conclusion on line 300-301 states “Functional forward stability indicator is a good predictor of fear of falling in group of elderly people.” – The manuscript should change its title to reflect this conclusion, so that it is about predictors of fear of falling rather than actual falls. The reader might misinterpret this and think that prospective falls are investigated  Answer:  We completely agree with reviewer comment. So as we have mentioned before, we have changed the title of the manuscript as follows: “Fall related measures in elderly and Parkinson’s disease”.  Reviewer comment 4  Line: Conclusion Line 224 “Existence of safety margin indicates deficits in balance.” – This single-sentence statement is unclear to me. Every human should have a safety margin, the question is how close to the safety margin our COG is displaced, and in turn might indicate deficits in balance.  Answer:  We do agree with this logic. However, we wanted to present other ideas connected with functional efficiency. We agree that a greater safety margin indicates more time to introduce and choose the strategy of balance. However, a greater safety margin decreases the range of the maximum forward lean, thereby decreasing functional movement. All subjects should explore as much as possible of the base of support. In our understanding, the safety margin is located between the first metatarsal phalangeal joint and the location of the COP while keeping the body in a leaning position. If the COP is located closer to this joint, the safety margin has a smaller area. We hope that the reviewer would agree with this rationale.  Reviewer comment 3  Also Line 224 “The greater safety margin is combined with greater postural instability (10).” If by safety margin you mean the margin between COG and LOS, this is not true for falls. Again, see Johansson et al. 2019 in Human Movement Science. Here, the greater the area of the center of pressure in relation to the LOS, the greater the risk for incident falls. This means that a greater safety margin was beneficial and reduced the |
| --- | --- |

|  | fall risk.  Answer:  In our study, smaller values of the range of maximum forward lean correlated with lower scores on some clinical tests (the BBS, FR test, and Tinetti test) and with higher scores on the TUG test. This clearly means that lower FFSI values indicate a greater risk of falling. Furthermore, a smaller range of maximum forward lean results in a greater safety margin  Reviewer comment 3  Line 281 – please be consequent with your citation method. Here you have switched to writing out the names and the year, and have not used the numbered reference system used in the rest of the manuscript.  Answer:  Thank you for pointing this out. All citations have been checked once again and we have made relevant correction. |
| --- | --- |
| Additional Information: |  |
| Question | Response |
| Financial Disclosure  Enter a financial disclosure statement that describes the sources of funding for the work included in this submission. Review the [submission guidelines](http://journals.plos.org/plosone/s/submission-guidelines#loc-financial-disclosure-statement) for detailed requirements. View published research articles from [PLOS ONE](http://journals.plos.org/plosone/) for specific examples.  This statement is required for submission and will appear in the published article if the submission is accepted. Please make sure it is accurate. | The study was supported by the National Center for Research and Development Grant under the program STRATEG MED III within the “VB-Clinic” project no.  STRATEGMED3/306011/1/NCBR/2017. |
| Unfunded studies  Enter: The author(s) received no specific funding for this work.  Funded studies  Enter a statement with the following details:   - Initials of the authors who received each award - Grant numbers awarded to each author - The full name of each funder - URL of each funder website - Did the sponsors or funders play any role in the study design, data collection and analysis, decision to publish, or preparation of the manuscript? - NO - Include this sentence at the end of your statement: The funders had no role in   study design, data collection and analysis, decision to publish, or preparation of the manuscript.   - YES - Specify the role(s) played. |  |
|  |  |

| * typeset |  |
| --- | --- |
| Competing Interests  Use the instructions below to enter a competing interest statement for this submission. On behalf of all authors, disclose any [competing interests](http://journals.plos.org/plosone/s/competing-interests) that could be perceived to bias this work—acknowledging all financial support and any other relevant financial or nonfinancial competing interests.  This statement will appear in the published article if the submission is accepted. Please make sure it is accurate. View published research articles from [PLOS ONE](http://journals.plos.org/plosone/) for specific examples. | The authors have declared that no competing interests exist. |
| NO authors have competing interests  Enter: The authors have declared that no competing interests exist.  Authors with competing interests  Enter competing interest details beginning with this statement:  I have read the journal's policy and the authors of this manuscript have the following competing interests: [insert competing interests here] |  |
| * typeset |  |
| Ethics Statement  Enter an ethics statement for this submission. This statement is required if the study involved:   - Human participants - Human specimens or tissue - Vertebrate animals or cephalopods - Vertebrate embryos or tissues - Field research   Write "N/A" if the submission does not require an ethics statement. | The research was accepted by the Institutional Ethics Committee Medical University in Warsaw number KB/28/2014 |

| General guidance is provided below. Consult the [submission guidelines](http://journals.plos.org/plosone/s/submission-guidelines#loc-guidelines-for-specific-study-types) for detailed instructions. Make sure that all information entered here is included in the Methods section of the manuscript. |  |
| --- | --- |
| Format for specific study types  Human Subject Research (involving human participants and/or tissue)   - Give the name of the institutional review board or ethics committee that approved the study - Include the approval number and/or a statement indicating approval of this research - Indicate the form of consent obtained (written/oral) or the reason that consent was not obtained (e.g. the data were analyzed anonymously)   Animal Research (involving vertebrate animals, embryos or tissues)   - Provide the name of the Institutional Animal Care and Use Committee (IACUC) or other relevant ethics board that reviewed the study protocol, and indicate whether they approved this research or granted a formal waiver of ethical approval - Include an approval number if one was obtained - If the study involved non-human primates, add additional details about animal welfare and steps taken to ameliorate suffering - If anesthesia, euthanasia, or any kind of animal sacrifice is part of the study, include briefly which substances and/or methods were applied   Field Research  Include the following details if this study involves the collection of plant, animal, or other materials from a natural setting:   - Field permit number - Name of the institution or relevant body that granted permission |  |
|  |  |
| Data Availability  Authors are required to make all data | Yes - all data are fully available without restriction |

| underlying the findings described fully available, without restriction, and from the time of publication. PLOS allows rare exceptions to address legal and ethical concerns. See the [PLOS Data Policy](http://journals.plos.org/plosone/s/data-availability) and [FAQ](http://journals.plos.org/plosone/s/data-availability#loc-faqs-for-data-policy) for detailed information.  A Data Availability Statement describing where the data can be found is required at submission. Your answers to this question constitute the Data Availability Statement and will be published in the article, if accepted. |  |
| --- | --- |
| Important: Stating ‘data available on request from the author’ is not sufficient. If your data are only available upon request, select ‘No’ for the first question and explain your exceptional situation in the text box. |  |
| Do the authors confirm that all data underlying the findings described in their manuscript are fully available without restriction? |  |
| Describe where the data may be found in full sentences. If you are copying our sample text, replace any instances of XXX with the appropriate details.   - If the data are held or will be held in a public repository, include URLs, accession numbers or DOIs. If this information will only be available after acceptance, indicate this by ticking the box below. For example: All XXX files   are available from the XXX database (accession number(s) XXX, XXX.).   - If the data are all contained within the manuscript and/or Supporting Information files, enter the following: All relevant data are within the manuscript and its Supporting Information files. - If neither of these applies but you are able to provide details of access elsewhere, with or without limitations, please do so. For example:   Data cannot be shared publicly because of [XXX]. Data are available from the | All relevant data are within the manuscript and its Supporting Information files. |

| XXX Institutional Data Access / Ethics Committee (contact via XXX) for researchers who meet the criteria for access to confidential data.  The data underlying the results presented in the study are available from (include the name of the third party and contact information or URL).  • This text is appropriate if the data are owned by a third party and authors do not have permission to share the data.  * typeset |  |
| --- | --- |
| Additional data availability information: |  |

Manuscript	[Click here to access/download;Manuscript;Manuscript.docx](https://www.editorialmanager.com/pone/download.aspx?id=26852228&guid=712875c6-0de7-4f4c-9115-0d643d5f5a61&scheme=1)

1 **Fall-related measures in elderly individuals and Parkinson’s disease subjects**
2 Justyna Michalska^1¶*^, Anna Kamieniarz^1¶^, Anna Brachman^1¶^, Wojciech Marszałek^1¶^, Joanna 3	Cholewa^2&^ Grzegorz Juras^1&^, Kajetan J. Słomka^1&^

4

5	^1^Institute of Sport Sciences, The Jerzy Kukuczka Academy of Physical Education, Katowice, 6	Poland

7	^2^ Institute of Physioterapy and Health Sciences, The Jerzy Kukuczka Academy of Physical 8	Education, Katowice, Poland

9

10

11 *** Corresponding author:**
12 email: j.michalska@awf.katowice.pl (JM)

# Abstract

Falls pose a serious problem in elderly and clinical populations. Most often, they lead to a loss of mobility and independence. They might also be an indirect cause of death. The aim of this study was to determine an objective predictor of the fear of falling and falls in elderly subjects (ESs) and Parkinson’s disease (PD) subjects. Thirty-two ESs were examined in this study, of whom sixteen were diagnosed with PD. The testing procedures comprised force plate measurements (limit of stability test–LOS test) and clinical tests (Berg Balance Scale,

Functional Reach Test, Timed Up and Go test, Tinetti test). The Falls Efficacy Scale International (FES-I) was used to evaluate the fear of falling. The range of the maximum forward lean was normalized to the length from the ankle joint to the head of the first metatarsal bone and was named the functional forward stability indicator (FFSI). The FFSI, derived from the LOS test, allowed us to demonstrate the real deficit in functional stability and individual safety margins. Moreover, the FFSI was highly correlated with the FES-I score and all clinical test results in elderly subjects (r>0,6; p<0.05). In PD subjects, the FFSI was poorly correlated with the fear of falling, the BBS score and the FR distance; however, a high correlation with the number of falls (r>0,6, p<0.05) was noted. The PD subjects presented a different balance strategy when close to their stability limits, which was also reflected in the lower values of sample entropy (t=(-2.40); p>0.05; d=0.87). The FFSI might be a good predictor of the fear of falling in the group of elderly people. Additionally, the FFSI allows us to show real balance deficits both in PD subjects and in their healthy peers without the need for a reference group and norms. In conclusion, it is postulated that the popular clinical assessments of postural balance in PD subjects should be accompanied by reliable posturography measurements.

# Introduction

Unintentional falls among people over 65 yr. of age are an increasing problem and are the leading cause of injury-related deaths (1). According to the WHO, between 2015 and 2050, the proportion of the world's population over 60 years old will nearly double, from 12% to 22%. Currently, in developed countries, the percentage of elderly subjects (ESs) is more than 20%. ESs experience falls due to significant deterioration of balance ability associated with a decrease in muscle strength, sensory function and widespread degeneration in the central nervous system (2).

Moreover, with age, accompanying neurological diseases may appear. Parkinson’s disease (PD) is the second most common neurodegenerative disorder (3). This chronic and progressive disease leads to rigidity, tremors, bradykinesia and thus impaired balance (4). Therefore, PD subjects are more exposed to the risk of falls than their peers (5).

The fear of falling (FOF) could appear well before and/or after an incident of a fall which creates a vicious cycle. Concomitant psychological symptoms of falls are the FOF, which is common among older adults regardless of whether they have sustained a fall. The FOF might also be a proper reaction to certain situations, leading elderly subject to be cautious, and can contribute to fall prevention through careful choice of movement activity. Nevertheless, ESs increasingly cease physical activity because of a FOF (6).

The FOF is assessed with many self-reported questionnaires, among which the Falls Efficacy Scale International (FES-I) test is widely used (7). It has become a useful instrument for predicting the risk of falls in elderly subjects due to its excellent reliability and validity across different populations (8). The questionnaire refers to most basic, everyday activities. Only people with a FOF may have difficulty performing these things.

Most daily activities require keeping the center of gravity (COG) within the confines of the base of support (BOS). Therefore, falls are quite frequent during everyday activities, especially when the COG is voluntarily shifted near or outside the limit of stability (LOS) (9). The LOS is depicted by the stability boundary and anatomically was assumed to be the foot envelope. The COG usually travels along a much smaller area of the BOS (10). It is suggested that the anterior mechanical stability boundary is located on the line connecting the extreme points located in the sagittal plane at the height of the first heads of the metatarsal bones (11). Moreover, from a biomechanical point of view, the rotation axis is located slightly further than the area of the first metatarsophalangeal joint (12). Crossing this mechanical limit inevitably involves changing the standing strategy (heel rise or toes co-contraction) or imposing gait initiation.

In older adults over 65 yr, 60% of falls occur in a forward direction (13). Additionally, Youn et al. (14) noticed that more than 70% of PD subjects had a history of falls in the forward direction. Therefore, in the present study, we focused on exploring the anterior stability boundary. The most frequent measure used to identify the stability boundary is the limit of stability (LOS) test (15). During this test, the detachment of the heel from the ground is prohibited. The calculation of the maximum forward lean range with respect to the mechanical (anatomical) stability boundary should allow subjects to reach the boundary that is in close proximity to the real safety margin. It is crucial for appropriate assessment of balance instability (11).

Clinical assessment of the risk of falling is based on the Berg Balance Scale (BBS), the Tinetti test or the Timed Up and Go (TUG) test. These tests aim to evaluate the integration of results of the systems involved in postural stability. Unfortunately, clinical tests do not facilitate the understanding of the etiology of fall-related concerns (16). Clinical tests are often based on a qualitative assessment (BBS, Tinetti test). Despite the clear assessment instructions, the researcher may influence the test results. We postulate more specific and reliable tools to be used for the diagnosis and comprehension of complex postural control processes (17). With an inefficient postural strategy (ankle and hip strategy) in elderly subjects, falls usually occur during movements where the COG is shifted near or outside the BOS (18). It seems that the LOS test is the most appropriate measurement. This is consistent with Johansson et al. (19) report which shows that postural sway in relation to assisted stability limits appears to be a valid predictor of incident falls. Additionally, posturography allows one to assess the difficulty of the motor task by analyzing COP regularity. The regularity of COP trajectories is quantified by sample entropy (SampEn). This was found to be positively related to the degree of attention invested in postural control (20). Higher values of SampEn indicate more irregularity in the COP signals. The basic assumption is that the automatic control processes increase the entropy of the signal while volitional control decreases the signal (21). In a certain sense, an analysis of SampEn should allow us to determine for which study group (ESs or PD subjects) the LOS test was more challenging.

The aim of this study was to determine an objective predictor of the fear of falling and falls in ESs and patients with neurodegenerative disorders. For this purpose, the clinical and laboratory tests were analyzed. Depending on the study groups, the proposed objective measure could replace or support clinical assessment. Posturographic examination can also register early and nonvisible postural control changes, so we assumed that this objective assessment might replace clinical tests among ESs. We also hypothesized that the identification of the real functional limit of support with the LOS test would allow us to assess not only the fear of falling but also the risk of falls. Neurological disorders lead to changes in the mechanism of postural control (4). We assumed that PD subjects present different balance strategies when their COG is located near their stability boundary. The analysis of the SampEn should allow us to identify these changes.

# Methods

## Subjects

Sixteen PD subjects and sixteen anthropometrically matched healthy controls voluntarily participated in the study (Table 1). PD subjects were recruited from the Local Association of Parkinson's disease. The research took place at the Academy of Physical Education in Katowice. Participants were males and females. A preliminary intragroup comparison with respect to the analyzed variables revealed that sex did not differentiate the groups. Based on a medical interview, the general inclusion criteria for PD subjects were age ≥

65 years, identified and diagnosed Parkinson’s disease, and stage III disease according to the Hoehn and Yahr scale. The exclusion criteria were as follows: no consent to participate in the research; dementia or cognitive impairment (MMSE score below 26 points); and neuromuscular, vestibular or orthopedic disorders. PD subjects were tested during the “ON period” of their usual antiparkinsonian medication (1 h after taking their usual dose of medication). The subjects gave informed written consent for voluntary participation in the study. The research was approved by the Institutional Ethics Committee of the Medical

University of Warsaw (number KB/28/2014).

*Table 1. Characteristics of subjects*

| Elderly subjects | | | | Parkinson’s disease subjects | | | |
| --- | --- | --- | --- | --- | --- | --- | --- |
|  | Sample size | | 8M/8F | 9M/7F | | |  |
|  | Age [years] | | 67 (65-69) | 65 (65-69.5) | | |  |
|  | Height [cm] | | 163.8 (160.8-166.8) | 167 (165.2-174.2) | | |  |
|  | Weight [kg] | | 74.8 (68.07-81.6) | 77.9 (71.6-84.2) | | |  |
|  | FES-I (score) | | 11.4 (8.4-14.27) | 12.3 (9.3-15.2) | | |  |
|  | Low concern | | n=5 (31.25%) | n=7 (43.75%) | | |  |
|  | Moderate concern | | n=7 (43.75%) | n=3 (18.75%) | | |  |
|  | High concern | | n=4 (25%) | n=6 (37.5) | | |  |
|  | Fellers | | n=3 (18.75%) | n=6 (37.5%) | | |  |
|  | FFSI (%) | | 82.78 (77.7-87.9) | 81.00 (74.2-87.9) | | |  |
|  | BBS (score) | **54.4 (53.3-55.5)** | | | **47.6 (41.4-55.4)** |  |  |
|  | FR (cm) | 27 (24.6-29.3) | | | 22 (17.6-28) |  |  |
|  | Tinetti test (score) | 27.5 (27.2-27.8) | | | 23 (19.9-26.6) |  |  |
|  | TUG (s) | **7.14 (6.5-7.7)** | | | **10.28 (8.1-13.5)** |  |  |

*Abbreviations: variables presented by mean values and 95% confidence intervals (CI), F, female; M: male FES-I, Falls Efficacy Scale International; FFSI, Functional Forward Stability Indicator; BBS, Berg Balance Scale; FR, Functional Reach; TUG, Timed Up and Go; bold indicate a statistically significant differences between group p<0.05*

## Apparatuses and procedures

Functional stability was evaluated with a force platform (AMTI, Accugait, Watertown,

MA, USA). The anthropometrics of the foot were measured with a spreading caliper. The Falls Efficacy Scale International (FES-I) was used to evaluate the fear of falling. It includes 7 activities with four possible answers: not at all concerned (one point), somewhat concerned

(two points), fairly concerned (three points) and very concerned (four points). The highest score (very concerned about falling) is, therefore, 28, and the minimum (no concern about falling) score is 7. Additionally, subjects were asked about their history of falls, which was defined as

“*unintentionally coming to rest on the ground or a lower level”* during the preceding year (22)*.* Information about the cause and direction of falls as well as possible injury from falls was also collected. The LOS test was used to evaluate functional stability and was conducted as described in the study by Juras et al. (23). This test consists of three distinct phases. The 1st phase includes quiet standing (QS) and lasts 10 seconds. During the 2nd phase, after an auditory signal, subjects lean forward as quickly and far as they can. The 3rd phase is associated with maintaining the inclined position. The fixation point is placed 3 m away from the participants on the wall in front of them at eye level. The measurement was interrupted and repeated if subjects lifted their heels. The LOS test lasted 30 seconds and was repeated three times. The average values of posturographic variables of three repetitions were taken for further analysis.

The testing procedures also involved clinical tests widely applied for balance assessment in ESs and PD subjects, such as the BBS, Tinetti test, FR test and TUG test. In the first three tests, a higher score indicates better balance ability. A shorter time in the TUG test suggests a good result.

## Data processing

Raw platform data were processed offline using MATLAB (MathWorks Inc., Natick,

MA, USA, v. r2017b). A low-pass 4-order Butterworth filter with a sampling frequency of 7 Hz was used for forces (Fx, Fy, Fz) and moments (Mx, My, Mz), which were later used to calculate the center of foot pressure (COP). Further COP analysis was based on the anatomical foot characteristics. The platform sampling frequency was 100 Hz. The following variables of COP displacement were calculated for the 1st and 3rd phases of the LOS test: SampEn in an anterior-posterior (AP) and medio-lateral (ML) plane (24). In the 2nd phase of the LOS test, the range of the maximum forward lean was normalized to the length from the ankle joint to the head of the first metatarsal bone and was named the functional forward stability indicator (FFSI). The calculation of the forward functional stability indicator [%] (FFSI) was conducted as follows:

*Abbreviations NMVE – normalized maximal voluntary COP excursion range calculated from the position of the medial malleolus to the average COP in the third phase of the LOS test, corresponding to maintenance in the inclined position [cm],*

*FFL – the length of the forefoot [cm].*

## Statistical analysis

The basic parameters of the descriptive statistics were calculated and analyzed. The ShapiroWilk test was used to check the data for normal distribution. The parametric and nonparametric tests were used. An independent-sample t-test was conducted to compare the anthropometric variables FFSI and SampEn from ESs and PD subjects. The effect sizes for the main and interaction effects are reported as Cohen’s d. The Mann-Whitney U test was conducted to compare values of FES-I, BBS, Tinetti, FR and TUG test from ESs and PD subjects. Spearman’s rank test was used to evaluate the associations among the FFSI and FES-I, clinical test results, and the number of falls. The levels of correlation were considered weak positive (0 – 0.3), weak negative (-0.3 – 0), moderate positive (0.3 – 0.7), moderate negative (-0.3 – (-0.7)), high positive (0.7 – 1.0), and high negative (-0.7 – (-1.0)) (25). Intraclass correlation coefficients (ICCs) were calculated for the LOS test (FFSI, SampEn phase 1, SampEn phase 3). The levels of reliability were considered poor (ICC < 0.50), moderate (0.50 ≤ ICC < 0.75), good (0.75 ≤ ICC < 0.90), and excellent (ICC ≥ 0.90), according to Portney et al (2000) (26). The LOS test achieved excellent reliability with 3 repetitions. The level of significance was set at p ≤. 0.05. All calculations were carried out using STATISTICA v.13.1 (StatSoft, Inc., USA).

# Results

## The analysis of the limit of stability of the COP characteristics between the groups

There were no significant differences between ESs and PD subjects in the FFSI (t= (-0.44); p>0.05; d=0.16). However, ESs leaned further than PD. Additionally, during the 1st phase of the LOS test, there were no significant differences in the value of SampEn between the two groups (t= (-1.99); p>0.05; d=0.73). Significantly lower values of SampEn in the 3rd phase of the LOS test were observed in PD subjects than in ESs (t= (-2.40); p>0.05; d=0.87) (Fig 1).

**Fig 1. The median value of sample entropy in the anterior-posterior direction (minimum and maximum marked as error bars) in the first and third phases of the limit of stability**

**test.**

Abbreviations: ESs, elderly subjects; PD, Parkinson’s disease subjects

## Correlation between the FFSI and the fear and risk of falling in elderly subjects

A significant positive correlation was observed between the FFSI and clinical test results, such as the BBS, FR test and Tinetti (p< 0.05). There was also a significant negative correlation between the FFSI and the TUG test and FES-I scores (p< 0.05). The numbers of falls did not show any correlations with maximum forward lean as assessed by the FFSI (p> 0.05) (Table

2).

*Table 2. Spearman’s correlation coefficient between FFSI and balance clinical test in ESs and*

*PD subjects*

|  | Group | BBS | FR | Tinetti | TUG | FES-I | NoF |
| --- | --- | --- | --- | --- | --- | --- | --- |
| FFSI [%] | ES | **0,634** | **0,674** | **0,759** | **-0,874** | **-0,831** | -0,229 |
| PD 0,352 0,471 **0,698 -0,626** -0,480 **-0,604** | | | | | | | |

*Abbreviations: ESs, elderly subjects; PD, Parkinson’s disease subjects; FFSI, functional forward stability indicator; FES-I, Falls Efficacy Scale International; BBS, Berg Balance Scale; FR, Functional Reach; TUG, Timed Up and Go; NoF, number of falls; bold indicates a statistically significant correlation p<0.05.*

**Correlation between the FFSI and the fear and risk of falling in**

## Parkinson’s Disease subjects

There was a significant positive correlation between the FFSI and Tinetti test scores (p< 0.05).

Additionally, a significant negative correlation was observed between the FFSI and TUG test as well as the number of falls (p<0.05). BBS, FR test and FES-I scores did not show any correlations with maximum forward lean as assessed by the FFSI (p> 0.05) (Table 2).

## Correlation between clinical tests and the fear and risk of falling in elderly subjects and PD subjects

There was only one significant negative correlation between the fear of falling and the TUG test scores in the ES group (p< 0.05). The rest of the clinical test scores did not correlate with the FES-I or the number of falls. In contrast, in PD subjects, all clinical test scores were significantly negatively correlated with the FES-I (p< 0.05). Tinetti test scores showed a significant negative correlation with the number of falls (p< 0.05). TUG test significantly positively correlated with the number of falls (p< 0.05). There were no correlations between BBS or FR test and the number of falls (p> 0.05) (Table 3).

*Table 3. Spearman’s correlation coefficients between among balance clinical test scores, the FES-I score, and the number of falls in elderly subjects and PD subjects*

| Group | Balance clinical tests | FES-I | Number of falls |
| --- | --- | --- | --- |
| ES | BBS  FR | -0,481  -0,468 | -0,203  -0,313 |
|  | Tinettii | -0,421 | -0,199 |
|  | TUG | **0,703** | 0,134 |
| PD | BBS  FR | **-0,698**  **-0,554** | -0,404  -0,346 |
|  | Tinettii | **-0,858** | **-0,689** |
|  | TUG | **0,723** | **0,582** |

*Abbreviations: ESs, elderly subjects; PD, Parkinson’s disease subjects; FES-I, Falls Efficacy Scale International; BBS, Berg Balance Scale; FR, Functional Reach; TUG, Timed Up and*

*Go; bold indicate a statistically significant correlation p<0.05.*

# Discussion

The FR test and the LOS test are the most frequent measures of functional balance, suggested by Clark et al. (27) to be complementary due to the differences in task constraints. Nevertheless, neither of them differentiated PD subjects from their healthy peers. Similar results were reported by Ryckewaert et al.(28) and Mancini et al. (29). On the other hand, significant differences between PD subjects and their healthy peers were presented by Behrman et al. (30), where the authors observed a significantly lower range of FR distance in PD subjects in comparison to the control group. They have also found that FR test scores below 25 cm indicate a high risk of falls. Based on this we would qualify our PD subjects, with their functional reach distance of 22,8 cm, as the at-risk group, even though there were no significant differences between the study groups. The latter might suggest that the LOS test is less valuable than the FR test. However, it is postulated that the range of maximum forward lean calculated with respect to the anatomical stability boundary allows us to demonstrate the real deficit of functional stability (31), which we believe is very important information with high practical value.

ESs used 82.78% of their functional BOS, which is located between the head of the first metatarsal bone and the medial malleolus (ankle joint). Their safety margin equaled 17.22%, in comparison to 19% for PD subjects. So PD subjects used a smaller area of the functional BOS during the LOS test than ESs (Fig 2). A greater safety margin decreases the range of the maximum forward lean, thereby decreasing functional movement. So the existence of a safety margin might indicate deficits in balance, thus our research hypothesis was validated.

According to Słomka et al. (31), a safety margin is located between the first metatarsal phalangeal joint and the COP location while keeping the body in a leaning position. Therefore, the lower range of the maximum forward lean increases the area of the safety margin. A greater safety margin is combined with greater postural instability (11). It is obvious that appropriate rehabilitation can improve balance (32). Therefore, purposeful therapy should be aimed at reducing the safety margin and allowing the relocation of the COP near the anatomical stability boundary during the LOS test. The FFSI bears clear information about functional stability deficits in the sagittal plane, and it also indicates a target value in the range of maximum forward COP excursion for patients in balance rehabilitation.

**Fig 2. Forward stability region**

Abbreviations: ESs, elderly subjects; PD, Parkinson’s disease subjects; FFSI, functional

forward stability indicator; FASL, forward anatomical stability limit; MS, margin safety

In the present study, the FFSI was highly correlated with the FOF in the control group. The decreased range of maximum forward lean indicated an increased postural threat. In turn, there were no correlations between FR test scores and both the FOF and the history of falls in healthy ESs. The LOS test is more specific than the FR test because it stresses the use of the ankle strategy (33), which minimizes early and small perturbations of upright posture. Different movement strategies adopted in FR test may significantly affect the final result and include the rotation of the trunk and a combination of the ankle and hip strategy (34). The LOS test is less prone to such strategy alterations during performance. We also confirmed our hypothesis that the LOS test might replace FR test in the diagnosis of ESs, which leads to conclusion that among healthy ESs the computerized posturography should be considered a more appropriate measure for the early identification of balance deficits than subjective tests and FR test.

Other clinical balance test scores as well as FR test are poor predictors of the fear of falling in groups of healthy ESs. In the present study, only TUG test were correlated with FESI scores. Neither of them were correlated with a history of falls. Landers et al. (35) showed which self-reported questionnaires are most clinically applicable to the prediction of falls in the elderly population. They also indicated that TUG test is the most predictive clinical test of the risk of falls. In general, clinical tests detect visible balance deficits. However, the identification of postural control changes that are invisible to the naked eye should be pursued. The FFSI allows the detection of the area of the safety margin, and it is correlated with the FOF in healthy

ESs. Moreover, the FFSI is also highly correlated with clinical test scores, such as TUG and Tinetti test scores, which are known to be good predictors of the risk of falls (2). We can conclude that based on our results, it is not necessary to use the whole battery of clinical tests for balance assessment to achieve satisfactory diagnostic outcomes, and our research hypothesis was validated. Acquiring more information in less time is especially important when conducting diagnoses in specific populations and situations.

The diagnosis of the PD subjects led to different observations. According to the Hoehn and Yahr scale, all PD subjects in this study were in stage III. Everyone had visible and persistent changes in postural control (3), which could be detected by both clinical tests and posturography measurements. All clinical test scores were highly correlated with the FOF.

Additionally, the Tinetti and TUG test were highly correlated with a history of falls. Nevertheless, the FFSI had a poor correlation with the FOF, but was highly correlated with a history of falls. Moreover, the FFSI was associated with only two clinical tests of the risk of falls. Our results validated our research hypothesis; therefore we postulate that balance assessments of the PD population should be made both with clinical tests and with computerized posturography. Clinical tests also assess movements such as walking forward, quiet standing and sitting down, which are indicated by Robinovitch et al. (36) as the most risk of falling activities.

As shown, the differences between PD subjects and their healthy peers can be easily detected with BBS and TUG tests. Whereas the TUG test is an incontestably appropriate measure to assess the dynamic balance among PD subjects, using the BBS for dynamic balance assessment is more questionable (37). Some authors indicated that the BBS, when used to measure balance in patients with PD, may not completely assess the whole spectrum of postural control impairments typical for this disease (38). Moreover, La Porta et al. (39) suggested that the BBS, even if modified by the new rating scale, may not be an effective tool for the measurement of early postural control disturbance in patients with PD.

Although there were no differences between PD subjects and the control group, it could be observed that the LOS test was more demanding for PD subjects. During the 3rd phase of the test, where subjects had to maintain an inclined position, PD subjects were characterized by lower values of SampEn. According to Richman and Moorman (2000) (24), low values of entropy show more regularity in the COP signal. Therefore, less chaotic excursions may be defined as a characteristic of an unsuccessful vigilant strategy to maintain balance. Furthermore, Donker et al. (40) showed that the amount of attention invested in keeping an upright posture is positively correlated with sway regularity. Based on these reports, PD subjects paid more attention to maintaining an inclined position than the control group (Fig 1). Therefore, we confirmed our research hypothesis that the LOS test is a more challenging motor task for PD subjects. Reinert et al. (41) confirmed that the use of nonlinear analysis methods such as SampleEn may improve the ability of the LOS test to identify differences in performance that are not currently revealed by traditional sway ranges.

This study has several limitations. A larger sample size would have been better as it would help to better generalize the results. Additionally, it is known that psychological factors, which had not been taken into account in this study, are associated with increased postural threat and could also affect the processing of postural control. Finally, it is known that pharmacological treatments may have an impact on postural control in PD subjects. Although we did our best to satisfy the standardization of procedures, we did not explore the problem of the impact of medication on postural control. Future research should be focused on the prospective risk of falls. It will be useful to create FFSI norms that can help diagnose the risk of falls.

# Conclusion

The present study highlights the importance of a quantitative approach for balance evaluation in ESs and elderly subjects with neurological disorders. The functional forward stability indicator is a good predictor of the fear of falling in the group of elderly people. Additionally, the FFSI allows us to show real balance deficits both in PD subjects and in their healthy peers without using an external reference group or created norms. A complete assessment of postural balance in PD subjects should be made both with posturography measurements and with clinical tests.

**Conflict of interest statement**

All authors confirm that there are no known conflicts of interest.

# Bibliography

1. Haagsma JA, Olij BF, Majdan M, Beeck EF Van, Vos T, Castle CD, et al. Falls in older aged adults in 22 European countries : incidence , mortality and burden of disease from 1990 to 2017. Inj Prev. 2020;1–8.
2. Ambrose AF, Paul G, Hausdorff JM. Risk factors for falls among older adults: A review of the literature. Maturitas. 2013;75(1):51–61.
3. Lee HK, Altmann LJP, McFarland N, Hass CJ. The relationship between balance confidence and control in individuals with Parkinson’s disease. Park Relat Disord.

2016;26:24–8.

4. Tangen GG, Bergland A, Engedal K, Mengshoel AM. The importance of parkinsonian signs for gait and balance in patients with Alzheimer’s disease of mild degree. Gait & Posture . 2017;51:159–61.
5. Stolze H, Klebe S, Zechlin C, Baecker C, Friege L, Deuschl G. Falls in frequent neurological diseases: Prevalence, risk factors and aetiology. J Neurol.

2004;251(1):79–84.

6. Park JH, Cho H, Shin JH, Kim T, Park SB, Choi BY, et al. Relationship among fear of falling, physical performance, and physical characteristics of the rural elderly. Am J Phys Med Rehabil. 2014;93(5):379–86.
7. Del-Río-Valeiras M, Gayoso-Diz P, Santos-Pérez S, Rossi-Izquierdo M, FaraldoGarcía A, Vaamonde-Sánchez-Andrade I, et al. Is there a relationship between short FES-I test scores and objective assessment of balance in the older people with ageinduced instability? Arch Gerontol Geriatr. 2016;62:90–6.
8. Kempen GIJM, Todd CJ, Van Haastregt JCM, Zijlstra GAR, Beyer N, Freiberger E, et al. Cross-cultural validation of the Falls Efficacy Scale International (FES-I) in older people: Results from Germany, the Netherlands and the UK were satisfactory. Disabil Rehabil. 2007;29(2):155–62.
9. Maki BE, McIlroy WE. Control of rapid limb movements for balance recovery: Agerelated changes and implications for fall prevention. Age Ageing.

2006;35(SUPPL.2):12–8.

10. Forth KE, Fiedler MJ, Paloski WH. Gait & Posture Estimating functional stability boundaries for bipedal stance. Gait Posture. 2011;33(4):715–7. Available from: http://dx.doi.org/10.1016/j.gaitpost.2010.12.024
11. Błaszczyk JW., Lowe DL.. Ranges of postural stability and their changes in the elderly.

Gait Posture. 1994;2(November):11–7.

12. Chan CW, Rudnis A. Foot Biomechanics During Walking and Running. Mayo Clin

Proc. 1994;69(5):448–61.

13. O’Neill TW, Varlow J, Silman AJ, Reeve J, Reid DM, Todd C, et al. Age and sex influences on fall characteristics. Ann Rheum Dis. 1994;53(11):773–5.
14. Youn J, Okuma Y, Hwang M, Kim D, Cho JW. Falling Direction can Predict the

Mechanism of Recurrent Falls in Advanced Parkinson’s Disease. Sci Rep.

2017;7(1):7–12.

15. Duarte M, Zatsiorsky VM. Effects of body lean and visual information on the equilibrium main- tenance during stance. Exp Brain Res. 2002;60–9.
16. Pauelsen M, Vikman I, Johansson Strandkvist V, Larsson A, Röijezon U. Decline in sensorimotor systems explains reduced falls self-efficacy. J Electromyogr Kinesiol.

2018;42(May):104–10.

17. Kamieniarz A, Michalska J, Brachman A, Pawłowski M, Słomka KJ, Juras G. A posturographic procedure assessing balance disorders in parkinson’s disease: A systematic review. Clin Interv Aging. 2018;13:2301–16.
18. Cleworth TW, Inglis JT, Carpenter MG. Postural threat influences the conscious perception of body position during voluntary leaning. Gait Posture. 2018;66(April):21–

5.

19. Johansson J, Jarocka E, Westling G, Nordström A, Nordström P. Predicting incident falls: Relationship between postural sway and limits of stability in older adults. Hum Mov Sci. 2019;66(October 2018):117–23.
20. Stins JF, Michielsen ME, Roerdink M, Beek PJ. Sway regularity reflects attentional involvement in postural control: effects of expertise, vision and cognition. Gait

Posture. 2009 Jul [cited 2015 Apr 28];30(1):106–9. Available from:

21. Roerdink M, Hlavackova P, Vuillerme N. Center-of-pressure regularity as a marker for attentional investment in postural control: a comparison between sitting and standing postures. Hum Mov Sci. 2011 Apr; 30(2):203–12.
22. Jean-Baptiste Mignardot, Olivier Beauchet CACC, Deschamps T. Postural sway, falls, and cognitive status: a cross-sectional study among older adults. J Alzheimers Dis.

2014;41(2):431–9.

23. Juras G, Słomka K, Fredyk A, Sobota G, Bacik B. Evaluation of the Limits of Stability (LOS) Balance Test. J Hum Kinet. 2008;19(1):39–52.
24. Richman JS, Moorman JR. Physiological time-series analysis using approximate entropy and sample entropy. AmJ Physiol Hear CircPhysiol. 2000;278(03636135):H2039–49.
25. Ratner B. The correlation coefficient: Its values range between 1/1, or do they. J

Targeting, Meas Anal Mark. 2009;17(2):139–42.

26. Portney LG WM. Foundations of Clinical Research: Applications to Practice. Upper

Saddle River, NJ: Prentice Hall Health; 2000.

27. Clark S, Iltis PW, Anthony CJ, Toews A. Comparison of Older Adult Performance

During the Functional-Reach and Limits-of-Stability Tests. J Aging Phys Act. 2005;13:266–75.

28. Ryckewaert G, Luyat M, Rambour M, Tard C, Noël M, Defebvre L, et al. Selfperceived and actual ability in the functional reach test in patients with Parkinson’s disease. Neurosci Lett. 2015;589:181–4.
29. Mancini M, Rocchi L, Horak FB, Chiari L. Effects of Parkinson’s disease and levodopa on functional limits of stability. Clin Biomech. 2008 May;23(4):450–8.
30. Behrman AL, Light KE, Flynn SM, Thigpen MT. Is the functional reach test useful for identifying falls risk among individuals with Parkinson’s disease? Arch Phys Med Rehabil. 2002;83(4):538–42.
31. Słomka KJ, Michalska J, Marszałek W, Bacik B, Juras G. Forward functional stability

indicator (FFSI) as a reliable measure of limits of stability. MethodsX. 2020;7:10–6. 32.	Lesinski M, Hortobagyi T, Muehlbauer T, Gollhofer A, Granacher U. Effects of Balance Training on Balance Performance in Healthy Older Adults: A Systematic Review and Meta-analysis. Sport Med. 2015;45(12):1721–38.

33. Horak FB. Postural orientation and equilibrium: What do we need to know about neural control of balance to prevent falls? Age Ageing. 2006;35(SUPPL.2):7–11.
34. Kage H, Okuda M, Nakamura I, Kunitsugu I, Sugiyama S, Hobara T. Measuring

Methods for Functional Reach Test: Comparison of 1-Arm Reach and 2-Arm Reach.

Arch Phys Med Rehabil. 2009;90(12):2103–7

35. Landers MR, Oscar S, Sasaoka J, Vaughn K. Balance Confidence and Fear of Falling Avoidance Behavior Are Most Predictive of Falling in Older Adults: Prospective

Analysis. Phys Ther. 2015;96(4):433–42.

36. Robinovitch SN, Feldman F, Yang Y, Schonnop R, Leung PM, Sarraf T, et al. Video capture of the circumstances of falls in elderly people residing in long-term care : an observational study. 2010;47–54.
37. Boulgarides LK, Mcginty SM, Willett JA, Barnes CW. Use of Clinical and

Impairment-Based Tests to Predict Falls by Community-Dwelling Older Adults. Phys Ther. 2003;83(4):328–39.

38. King LA, Priest KC, Salarian A, Pierce D, Horak FB. Comparing the Mini-BESTest with the Berg Balance Scale to evaluate balance disorders in Parkinson’s disease.

Parkinsons Dis. 2012;2012.

39. La Porta F, Giordano A, Caselli S, Foti C FF. Is the Berg Balance Scale an effective tool impairments in patients with Parkinson’s disease for the measurement of early postural control Evidence from Rasch analysis. Eur J Phys Rehabil Med. 2015;51:705–

16.

40. Donker SF, Ledebt A, Roerdink M, Savelsbergh GJP, Beek PJ. Children with cerebral palsy exhibit greater and more regular postural sway than typically developing children. Exp Brain Res. 2008;184(3):363–70.
41. Reinert SS, Kinney AL, Jackson K, Diestelkamp W, Bigelow K. Age stratification and sample entropy analysis enhance the limits of stability tests for older adults. J Appl

Biomech. 2017;33(6):419–23.

# 466 Supporting information

467	**S1 Data sheet**

Figure 1	[Click here to access/download;Figure;Figure 1.tif](https://www.editorialmanager.com/pone/download.aspx?id=26782782&guid=f87b1def-7bfd-42b9-9d93-aade062a5eb9&scheme=1)

Figure 2	[Click here to access/download;Figure;Figure 2.tif](https://www.editorialmanager.com/pone/download.aspx?id=26782783&guid=5fde4c19-2415-4519-ab45-2c3974b28f9b&scheme=1)

data sheet

[Click here to access/download](https://www.editorialmanager.com/pone/download.aspx?id=26852232&guid=a3ca9a4c-45ce-4389-a6a3-654ff2633d6c&scheme=1)

[Supporting Information data sheet.csv](https://www.editorialmanager.com/pone/download.aspx?id=26852232&guid=a3ca9a4c-45ce-4389-a6a3-654ff2633d6c&scheme=1)

Revised Manuscript with Track Changes

1 **~~Predictive~~Fall-related measures ~~of falls~~ in elderly individuals and Parkinson’s**
2 **~~Disease~~disease subjects**
3 Justyna Michalska^1¶*^, Anna Kamieniarz^1¶^, Anna Brachman^1¶^, Wojciech Marszałek^1¶^, Joanna 4	Cholewa^2&^ Grzegorz Juras^1&^, Kajetan J. Słomka^1&^

5

6 ^1^Institute of Sport Sciences, The Jerzy Kukuczka Academy of Physical Education, Katowice,
7 Poland

8	^2^ ~~Department~~Institute of ~~Physiotherapy~~Physioterapy and Health Sciences, The Jerzy 9	Kukuczka Academy of Physical Education, Katowice, Poland

10

11

12 *** Corresponding author:**
13 ~~Justyna Michalska PhD~~
14 ~~Institute of Sport Sciences~~
15 ~~The Jerzy Kukuczka Academy of Physical Education~~
16 ~~in Katowice, Poland~~
17 ~~tel.: +4832 207 51 60~~
18 email: j.michalska@awf.katowice.pl
19 (JM)

# Abstract

Falls pose a serious problem in elderly and clinical populations. Most often, they lead to a loss of mobility and independence. They might also be ~~as well~~ an indirect cause of death. The aim of this study was to determine ~~predictive measures of fall~~ an objective predictor of the fear of falling and falls in elderly subjects (ESs) and Parkinson’s ~~Disease patients.~~disease (PD) subjects. Thirty-two elderly subjects were examined in this study ~~from which~~, of whom sixteen were diagnosed with PD. The testing procedures comprised ~~of~~ force plate measurements (limit of stability test–LOS test) and clinical tests~~:~~ (Berg Balance Scale, Functional Reach Test, ~~Time~~Timed Up and ~~GO~~Go test, Tinetti ~~Test.~~test). The Falls Efficacy Scale International (FESI) was used to evaluate the fear of falling. The range of the maximum forward lean was normalized to the length from the ankle joint to the head of the first metatarsal bone and ~~it~~ was named the functional forward stability indicator (FFSI). The FFSI, derived from the LOS test, allowed us to demonstrate the real deficit in functional stability and individual safety margins. Moreover, the FFSI was highly correlated with the FES-I score and all clinical ~~tests~~test results in elderly subjects (r>0,6; p<0~~,~~.05). In PD subjects, the FFSI was poorly correlated with the fear of falling, the Berg Balance Scale score and the Functional Reach ~~test,~~distance; however, a high correlation with the number ~~the~~of falls (r>0,6, p<0~~,~~.05) was noted. The PD subjects presented a different balance strategy when ~~closing~~close to their stability limits, which was also reflected in the lower values of sample entropy (t= (-2~~,~~.40); p>0.05; d=0~~,~~.87). The FFSI ~~occurred to~~might be a good predictor of the fear of falling in the group of elderly people. Additionally, ~~it~~the FFSI allows us to show real balance deficits both in PD subjects and in their healthy peers without the need for a reference group and norms. In conclusion, it is postulated that the popular clinical ~~assessment~~assessments of postural balance in PD subjects should be accompanied by reliable posturography measurements.

# Introduction

Unintentional falls among people over 65 ~~years old is~~yr. of age are an increasing problem~~, being~~ and are the leading cause of injury-related deaths (1). According to the WHO, between 2015 and 2050, the proportion of the world's population over 60 years old will nearly double, from 12% to 22%. Currently, in ~~the~~ developed countries, the percentage of elderly subjects (~~ES) come to~~ESs) is more than 20%. ~~The ES~~ESs experience falls due to significant deterioration of balance ability associated with a decrease ~~of~~in muscle strength, sensory function and widespread degeneration in the central nervous system~~.~~ (2).

Moreover, with age, accompanying neurological diseases may appear. Parkinson’s ~~Disease~~disease (PD) is the second most common neurodegenerative disorder (~~2~~3). This chronic and progressive disease leads to rigidity, ~~tremor~~tremors, bradykinesia and thus impaired balance (~~3). So~~4). Therefore, PD ~~patients~~subjects are more exposed to the risk of falls than their peers (~~4~~5).

The fear of falling (FOF) could appear well before and/or after an incident of a fall which creates a vicious cycle. Concomitant psychological symptoms of falls are the FOF, which is ~~related to the subjective body imagination and body efficiency. It~~common among older adults regardless of whether they have sustained a fall. The FOF might also be a proper reaction to certain situations, leading ~~the~~ elderly subject to be cautious ~~behaviour~~, and can contribute to fall prevention through careful choice of movement activity. Nevertheless, ~~ES~~ESs increasingly cease physical activity because of ~~the~~a FOF (~~5~~6).

The FOF is assessed with many self-reported questionnaires, among which the Falls Efficacy Scale International ~~test~~ (FES-I) test is widely used (~~6~~7). It has become a useful instrument for predicting the risk of falls in ~~the~~ elderly subjects due to its excellent reliability and validity across different populations (~~7~~8). The questionnaire refers to ~~the~~ most ~~of the~~ basic, everyday activities ~~presented by disabled~~. Only people ~~would be likely to~~with a FOF may have difficulty ~~with because of their FOF.~~ performing these actions.

Most daily activities require keeping the center of gravity (COG) within the confines of the base of support (BOS). Therefore, ~~the~~ falls are quite frequent during everyday activities, especially when the COG is voluntarily shifted near or outside the limit of stability (~~8). Limit of stability~~LOS) (9). The LOS is depicted by the stability boundary and anatomically was assumed to be the foot envelope. ~~But the~~The COG usually travels along a much smaller area of the BOS (~~9~~10). It is suggested that the anterior mechanical stability boundary is located on the line connecting the extreme points located in the sagittal plane at the height of the first heads of the metatarsal bones (~~10~~11). Moreover, from a biomechanical point of view, the rotation axis is located slightly further than the area of the first metatarsophalangeal joint (~~11~~12). Crossing this mechanical limit inevitably involves ~~change~~ ~~of~~changing the standing strategy (heel rise or toes co-contraction) or ~~impose~~imposing gait initiation.

In older adults over 65 yr, 60% of falls occur in a forward direction (13). Additionally, Youn et al. (14) noticed that more than 70% of PD subjects had a history of falls in the forward direction. Therefore, in the present study, we focused on exploring the anterior stability boundary. The most frequent measure used to identify the stability boundary is the limit of stability (LOS) test (~~LOS test) (12~~15). During this test ~~a~~, the detachment of the heel from the ground is prohibited. The calculation of the maximum forward lean range with respect to the mechanical (anatomical) stability boundary should allow subjects to ~~demonstrate~~reach the boundary that is in close proximity to the real safety margin. It is crucial for appropriate assessment of ~~fear and risk of falling.~~balance instability (11).

Clinical assessment of the risk of falling is based on the Berg Balance Scale (BBS), the Tinetti ~~Test~~test or ~~Time~~the Timed Up and Go (TUG) test. These tests aim to evaluate the integration of results of the systems involved in postural stability. Unfortunately, clinical tests do not facilitate the understanding ~~the aetiology~~ of the ~~falls~~etiology of fall-related concerns (~~13).~~16). Clinical tests are often based on a qualitative assessment (BBS, Tinetti test). Despite the clear assessment instructions, the researcher may influence the test results. We postulate more specific and reliable tools to be used for the diagnosis and comprehension of ~~the~~ complex postural control processes (~~14). Since~~17). With an inefficient postural strategy

(ankle and hip strategy) in elderly subjects, falls usually occur during movements where the COG is shifted near or outside the BOS~~, it~~ (18). It seems that the LOS test~~,~~ is the most appropriate measurement. This is consistent with Johansson et al. (19) report which shows that postural sway in relation to assisted stability limits appears to be a valid predictor of incident falls. Additionally, posturography allows us to assess the difficulty of the motor task by analyzing COP regularity. The regularity of COP trajectories was quantified by the sample entropy (SampEn). This was found to be positively related to the degree of attention invested in postural control (20). Higher values of SampEn indicate more irregularity in the COP signals. The basic assumption is that the automatic control processes increase the entropy of the signal while volitional control decreases the signal (21). In a certain sense, an analysis of SampEn should allow us to determine for which study group (ESs or PD subjects) the LOS test was more challenging.

The aim of this study was to determine ~~predictive measures of fall in ES~~an objective predictor of the fear of falling and falls in ESs and patients with neurodegenerative disorders. For this purpose, the clinical and laboratory tests were ~~analysed.~~analyzed. Depending on the study groups, the proposed objective measure could replace or support clinical assessment. Posturographic examination can also register early and nonvisible postural control changes, so we assumed that this objective assessment might replace clinical tests among ESs. We also hypothesized that the identification of the real functional limit of support with the LOS test ~~allows~~ would allow us to assess not only the fear of falling but also the risk of falls. ~~The~~ ~~neurological~~Neurological disorders lead to changes in ~~a~~the mechanism of postural control

(4). We assumed that PD ~~patients~~subjects present different balance ~~strategy~~strategies when their COG is located near ~~to the~~their stability boundary. The analysis of the ~~sample~~ ~~entropy~~SampEn should allow us to identify these changes.

# Methods

## Subjects

Sixteen PD subjects and sixteen ~~age-~~anthropometrically matched healthy controls voluntarily participated in the study~~.~~ (S1 Table 1). PD subjects were recruited from the Local Association of Parkinson's disease. The research took place at the Academy of Physical Education in Katowice. Participants were males and females. A preliminary intragroup comparison with respect to the ~~analysed~~analyzed variables revealed that sex did not differentiate the groups. Based on a medical interview, the general inclusion criteria for PD ~~patients~~subjects were~~:~~ age ≥ 65 years, identified and diagnosed Parkinson’s ~~Disease,~~disease, and stage III disease according to the Hoehn and Yahr ~~Scale~~scale. The exclusion criteria were as follows: no consent to participate in the research~~,~~; dementia or cognitive impairment (MMSE score below 26 points)~~,~~; and neuromuscular, vestibular or orthopedic disorders. PD ~~patients~~subjects were tested during ~~„~~the “ON period” ~~at~~of their usual antiparkinsonian medication (~~one hour~~ 1 h after taking their usual dose of medication). The subjects gave informed written consent for voluntary participation in the study. The research was ~~accepted~~approved by the Institutional ~~Bioethics~~Ethics Committee~~.~~ of the Medical University of

Warsaw (number KB/28/2014).

*Table 1. Characteristics of subjects*

| Elderly subjects | | | | Parkinson’s disease ~~patients~~subjects | | | |
| --- | --- | --- | --- | --- | --- | --- | --- |
|  | Sample size | | ~~16~~8M/8F | ~~16~~9M/7F | |  |  |
|  | Age [years] | | 67 ~~± 3~~(65-69) | ~~63,8 ± 10,~~65 (65-69.5) | |  |  |
|  | Height [cm] | 163~~,~~.8 ~~± 5,5~~(160.8-166.8) | | | 167 ~~± 8,4~~(165.2-174.2) | |  |
|  | Weight [kg] | 74~~,~~.8 ~~± 12,8~~(68.07-81.6) | | | 77~~,~~.9 ~~± 11,8~~(71.6-84.2) | |  |
|  | FES-I (score) | 11~~,~~.4 ~~± 5,5~~(8.4-14.27) | | | 12~~,~~.3 ~~± 5,4~~(9.3-15.2) | |  |
|  | Low concern | n=5 (31.25%) | | | n=7 (43.75%) | |  |
|  | Moderate concern | n=7 (43.75%) | | | n=3 (18.75%) | |  |
|  | High concern | n=4 (25%) | | | n=6 (37.5) | |  |
|  | Fellers | n=3 (18.75%) | | | n=6 (37.5%) | |  |
|  | FFSI (%) | 82.78 (77.7-87.9) | | | 81.00 (74.2-87.9) | |  |
|  | BBS ~~*~~(score) | ~~56 (51-56)~~**54.4 (53.3-55.5)** | | | ~~52 (15-56~~**47.6 (41.4-55.4)** | |  |
|  | FR ~~*~~(cm) | 27 (~~18-37~~24.6-29.3) | | | ~~25 (5-36~~22 (17.6-28) | |  |
|  | Tinetti test ~~*~~(score) | 27~~,~~.5 (27~~-28~~.2-27.8) | | | ~~27 (8-28)~~23 (19.9-26.6) | |  |
|  | TUG ~~*~~(s) | **7**~~,24 (~~**.14 (6.5**~~,18-9,11~~**-7.7)** | | | ~~9,04 (~~**10.28 (8.1-13.5**~~,54-21~~**)** | |  |

*Abbreviations:* ~~* median (minimum and maximum)~~ *variables presented by mean values*~~,~~ *and*

*95% confidence intervals (CI), F, female; M: male FES-I,* *Falls Efficacy Scale International;*

*FFSI, Functional Forward Stability Indicator; BBS, Berg Balance Scale; FR, Functional Reach; TUG,* ~~Time~~*Timed Up and Go*~~.~~*; bold indicate a statistically significant differences between group p<0.05*

## ~~Apparatus~~Apparatuses and procedures

~~The functional~~Functional stability was evaluated with a force platform (AMTI, Accugait, Watertown, MA, USA). The anthropometrics of the foot were measured with a spreading ~~calliper~~caliper. The Falls Efficacy Scale International (FES-I) was used to evaluate the fear of falling. It includes 7 activities with four possible answers: not at all concerned (one point), somewhat concerned (two points), fairly concerned (three points) and very concerned (four points). The highest score (very concerned about falling) is, therefore, 28, and the minimum (no concern about falling) score is 7. Additionally, subjects were asked about their history of ~~the~~ falls, which was defined as “*unintentionally coming to rest on the ground or a lower level”* during the preceding year ~~(15)~~*~~.~~*(22)*.* Information about the cause~~,~~ and direction ~~and~~ of falls as well as possible injury from falls ~~were~~was also collected. The LOS test was used to evaluate functional stability and was conducted as described in the study by Juras et al. ~~(2008)~~

~~(16).~~(23). This test consists of ~~the~~ three distinct phases. The 1st phase includes quiet standing (QS) and ~~last~~lasts 10 seconds. During the 2nd phase, after an auditory signal, subjects lean forward as quickly and far as they can. The 3rd phase is associated with maintaining the inclined position. The fixation point is placed 3 m away from the participants on the wall in front of them at ~~the~~ eye level. The measurement was interrupted and repeated if subjects lifted their heels. The LOS test lasted 30 seconds and was repeated three times. The average values of posturographic variables of three repetitions were taken for further analysis.

The testing procedures also involved ~~also~~ clinical tests widely applied for ~~balanceassessmentt~~balance assessment in ~~the ES~~ESs and PD ~~patients~~subjects, such as the BBS, ~~Tinetii~~Tinetti test, FR test and TUG test. In ~~three~~the first three tests, a higher score indicates ~~a~~ better balance ability. A shorter time in the TUG test suggests a good result.

## Data processing

Raw platform data were processed offline using MATLAB (~~Mathworks~~MathWorks Inc., Natick, MA, USA, v. r2017b). A low-pass 4-order Butterworth filter with a sampling frequency of 7 Hz was used for forces (Fx, Fy, Fz) and moments (Mx, My, Mz), which were later used to calculate the center of foot pressure (COP). Further COP analysis was based on the anatomical foot ~~characteristic.~~characteristics. The platform sampling frequency was 100

Hz. The following variables of COP displacement were calculated for the 1st and 3rd phases of the LOS test: ~~sample entropy (~~SempEn~~)~~ in an anterior-posterior (AP) and medio-lateral (ML) plane ~~(17). In the 2nd phase of LOS test: the range of maximum forward lean was normalized~~ ~~to length from the ankle joint to the head of first metatarsal bone and it was named as a~~(24). In the 2nd phase of the LOS test, the range of the maximum forward lean was normalized to the length from the ankle joint to the head of the first metatarsal bone and was named the functional forward stability indicator (FFSI). The calculation of the forward functional stability indicator

[%]	(FFSI)	was	conducted	as follows:

*~~Figure 1. Functional forward stability indicator~~*

*Abbreviations NMVE – normalized maximal voluntary COP excursion range calculated from the position of the medial malleolus to the average COP in the third phase of the LOS test, corresponding to ~~the~~ maintenance in the inclined position [cm],*

*FFL – the length of the forefoot [cm].*

~~Statistic~~

## Statistical analysis

The basic parameters of the descriptive statistics were calculated and ~~analysed~~analyzed. The Shapiro-Wilk test was used to check the data for normal distribution. ~~The~~The parametric and nonparametric tests were used. An independent-~~samples~~sample t-test was conducted to compare the ~~data~~ anthropometric variables FFSI and SampEn from ~~ES~~ESs and PD

~~patients~~subjects. The effect sizes for the main and interaction effects are reported as Cohen’s d~~-Cohen~~. The ~~Spearman~~Mann-Whitney U test was conducted to compare values of FES-I, BBS, Tinetti, FR and TUG test from ESs and PD subjects. Spearman’s rank test was used to evaluate the ~~association between~~associations among the FFSI and FES-I, clinical ~~tests,~~ test results, and the number ~~and~~of falls. The levels of correlation were considered weak positive (0 – 0~~,~~.3), weak negative (-0~~,~~.3 – 0), moderate positive (0~~,~~.3 – 0~~,~~.7), moderate negative (-0~~,~~.3 – (0~~,~~.7)), high positive (0~~,~~.7 – 1~~,~~.0), and high negative (-0~~,~~.7 – (-1~~,~~.0)) ~~(18).~~ (25). Intraclass correlation coefficients (ICCs) were calculated for the LOS test (FFSI, SampEn phase 1, SampEn phase 3). The levels of reliability were considered poor (ICC < 0.50), moderate (0.50 ≤ ICC < 0.75), good (0.75 ≤ ICC < 0.90), and excellent (ICC ≥ 0.90), according to Portney et al (2000) (26). The LOS test achieved excellent reliability with 3 repetitions. The level of significance was set at p ≤. 0~~,~~.05. All calculations were carried out using STATISTICA v.13.1 (StatSoft, Inc., USA).

# Results

## The analysis of the limit of stability of the COP characteristics between the groups

There were no significant differences between ~~ES~~ESs and PD ~~patients~~subjects in the FFSI (t=

(-0~~,~~.44); p>0~~,~~.05; d=0~~,~~.16). However, ~~it could be observed that ES~~ESs leaned farther than PD.

~~Also~~Additionally, during the 1st phase of the LOS test, there were no significant differences in the value of SampEn between the two groups (t= (-1~~,~~.99); p>0~~,~~.05; d=0~~,~~.73). Significantly lower values of SampEn in the 3rd phase of the LOS test were observed in PD ~~compared to the~~ ~~ES~~subjects than in ESs (t= (-2~~,~~.40); p>0~~,~~.05; d=0~~,~~.87) (S1 fig 1).

**Figure** *~~2~~***1. The median value of sample entropy in the anterior-posterior direction (minimum and maximum marked as** *~~a~~* **error bars) in the first and third** *~~phase~~***phases of**

**the limit of stability test.**

Abbreviations*~~:: ES~~*: ESs, elderly subjects; PD, Parkinson’s disease *~~patients~~*subjects

## Correlation between the FFSI and the fear and risk of falling in elderly subjects

A significant positive correlation was observed between the FFSI and clinical test results, such as the BBS, FR test and ~~Tinnetti~~Tinetti (p< 0~~,~~.05). There was also a significant negative correlation between the FFSI and the TUG test~~,~~ and FES-I scores (p< 0~~,~~.05). The numbers ~~the~~of falls did not show any correlations with maximum forward lean as assessed by the FFSI (p> 0~~,~~.05) (~~tab.~~S2 Table 2).

*Table 2. Spearman’s correlation coefficient between FFSI and balance clinical test in* ~~ES~~*ESs and PD subjects*

|  | Group | BBS | FR | ~~Tinettii~~Tinetti | TUG | FES-I | NoF |
| --- | --- | --- | --- | --- | --- | --- | --- |
| FFSI [%] | ES | **0,634** | **0,674** | **0,759** | **-0,874** | **-0,831** | -0,229 |
| PD 0,352 0,471 **0,698 -0,626** -0,480 **-0,604** | | | | | | | |

*Abbreviations:* ~~ES~~*ESs, elderly subjects; PD, Parkinson’s disease* ~~patients~~*subjects; FFSI,* ~~Functional~~*functional forward stability indicator; FES-I,* *Falls Efficacy Scale International; BBS, Berg Balance Scale; FR, Functional Reach; TUG,* ~~Time~~*Timed Up and Go; NoF, number of falls; bold indicates a statistically significant correlation p<0.05.*

**Correlation between the FFSI and the fear and risk of falling in**

## Parkinson’s Disease ~~patients~~subjects

There was a significant positive correlation between the FFSI and Tinetti test scores (p< 0~~,~~.05). ~~Also~~Additionally, a significant negative correlation was observed between the FFSI and TUG test ~~and~~ as well as the number of ~~the~~ falls (p<0~~,~~.05). BBS, FR test and FES-I scores did not show any correlations with maximum forward lean as assessed by the FFSI (p> 0~~,~~.05) (~~tab.~~S2 Table 2).

**Correlation between clinical tests and the fear and risk of falling**

## in elderly subjects and PD ~~patients~~subjects

There was only one significant negative correlation between the fear of falling and the TUG test scores in ~~a~~the ES group ~~of ES~~ (p< 0~~,~~.05). The rest of the clinical test scores did not correlate with the FES-I ~~and a~~or the number of ~~the~~ falls ~~Oppositely~~. In contrast, in PD ~~patients~~subjects, all clinical ~~tests~~ test scores were significantly negatively correlated with the FES-I (p< 0~~,~~.05). ~~The~~ Tinetti test scores showed a significant negative ~~correlations~~correlation with ~~a~~the number of ~~the~~ falls (p< 0~~,~~.05). ~~The~~ TUG test significantly positively correlated with the number of ~~the~~ falls (p< 0~~,~~.05). There were no correlations between BBS~~,~~ or FR test and the number ~~the~~of falls (p> 0~~,~~.05~~).~~

~~Table~~) (SM 3 Table 3).

*Table 3. Spearman’s correlation* ~~coefficient~~*coefficients between among balance clinical* ~~tests~~ ~~and~~*test scores, the FES-I*~~,~~ *score, and the number* ~~and direction~~ *of falls in elderly subjects and PD subjects*

| Group | Balance clinical tests | FES-I | Number of falls |
| --- | --- | --- | --- |
| ES | BBS  FR | -0,481  -0,468 | -0,203  -0,313 |
|  | Tinettii | -0,421 | -0,199 |
|  | TUG | **0,703** | 0,134 |
| PD | BBS  FR | **-0,698**  **-0,554** | -0,404  -0,346 |
|  | Tinettii | **-0,858** | **-0,689** |
|  | TUG | **0,723** | **0,582** |

*Abbreviations:* ~~ES~~*ESs, elderly subjects; PD, Parkinson’s disease* ~~patients~~*subjects; FES-I,* *Falls Efficacy Scale International; BBS, Berg Balance Scale; FR, Functional Reach; TUG,* ~~Time~~*Timed Up and Go; bold indicate a statistically significant correlation p<0.05.*

# Discussion

The FR test and the LOS test are the most frequent measures of functional balance ~~assessing~~ ~~the risk of falls in the elderly population. However,~~ , suggested by Clark et al. ~~(2005) (19)~~ ~~suggested that~~ (27) to be complementary due to the differences in task constraints~~, make FR~~ ~~and LOS tests complementary. Therefore, in this study subjects performed both FR and LOS~~ ~~test.~~. Nevertheless, neither of them differentiated PD ~~patients~~subjects from their healthy peers. Similar results were reported by Ryckewaert et al~~. (2015~~.(28) and Mancini et al. ~~(2008) (20)~~ ~~who noticed no~~(29). On the other hand, significant differences ~~in forward COP displacement~~ ~~during FR test between PD patients in “ON period” and their control group~~

S~~ignificant differences~~ between PD subjects and their healthy peers were presented by Behrman ~~at~~et al. ~~(2002) (21). They~~(30), where the authors observed a significantly lower range of FR distance in PD ~~patients compared~~subjects in comparison to the control ~~groups. According to~~ ~~Behrman at a. (2002) result of~~group. They have also found that FR test scores below 25 cm ~~indicates~~indicate a high risk of ~~fall. Although, in~~falls. Based on this we would qualify our ~~study~~ ~~there were no statistical differences between PD subjects and healthy peers, based on Behrman~~ ~~et al. (2002) result, the group of PD~~ PD subjects, with their ~~result of 22,8 cm of~~ functional reach distance ~~can be qualified~~of 22,8 cm, as the at-risk group~~.~~

I~~t is difficult to find norms for~~, even though there were no significant differences between the ~~LOS test. Moreover, in the present study, it did not show differences between study groups,~~ ~~what~~study groups. The latter might suggest ~~to be~~ that the LOS test is less valuable than the FR test. However, it is postulated that the range of maximum forward lean calculated with respect to the anatomical stability boundary allows us to demonstrate the real deficit of functional stability (~~22). ES~~31), which we believe is very important information with high practical value. ~~E~~Ss used 82~~,~~.78% of their functional BOS, which is located between the head of the first metatarsal bone and the medial ~~malleoulus~~malleolus (ankle joint). Their safety margin ~~equalled~~equaled 17~~,~~.22~~% with respect~~%, in comparison to 19% ~~of~~for PD subjects. ~~They~~So PD subjects used a smaller area of the functional BOS during the LOS test ~~(~~than ESs (S2 fig~~. 3).~~ ~~Existence~~ 2). A greater safety margin decreases the range of the maximum forward lean, thereby decreasing functional movement. So the existence of a safety margin ~~indicates~~might indicate deficits in balance~~. The~~, thus our research hypothesis was validated. According to Słomka et al. (31), a safety margin is located between the first metatarsal phalangeal joint and the COP location while keeping the body in a leaning position. Therefore, the lower range of the maximum forward lean increases the area of the safety margin. A greater safety margin is combined with greater postural instability (~~10~~11). It is obvious~~,~~ that appropriate rehabilitation ~~improves~~can improve balance (~~23~~32). Therefore, purposeful therapy should be aimed ~~to~~ ~~reduce~~at reducing the safety margin and ~~allow to relocate~~allowing the relocation of the COP near the anatomical stability boundary during the LOS test. The FFSI bears clear information about functional stability deficits in the sagittal plane, and it also indicates a target value in the range of maximum forward COP excursion for patients in balance rehabilitation.

Figure ~~3~~2. Forward stability region

Abbreviations~~:: ES~~: ESs, elderly subjects; PD, Parkinson’s disease ~~patients~~subjects; FFSI, ~~Functional~~functional forward stability indicator; FASL, ~~Forward~~forward anatomical stability limit; MS, margin safety

C~~omputerized posturography should be considered a more appropriate measure of balance~~ ~~ability among healthy ES than subjective tests.~~ In the present study, the FFSI was highly correlated with the FOF in the control group. The decreased range of maximum forward lean indicated an increased postural threat. In turn, there were no correlations between FR test scores and both the FOF and the history of falls in healthy ~~ES.~~ESs. The LOS test is more specific than the FR test because it stresses the use of the ankle strategy (~~24). Thanks to this strategy the~~ ~~center of mass is repositioned by moving the whole body as a single-segment inverted~~ ~~pendulum by production of torque at the ankle (25). This~~ 33), which minimizes early and small ~~perturbation~~perturbations of upright posture ~~which is crucial with respect to efficient postural~~ ~~control. Furthermore, other studies have shown subjects exhibiting the same reaching distance~~ ~~in FR which adopt different~~. Different movement strategies ~~during the~~ adopted in FR test~~. These~~ ~~strategies~~ may significantly affect the final result and ~~includes~~include the rotation of the trunk and a combination of the ankle and hip strategy (~~26). Therefore, the~~34). The LOS test ~~being~~is less prone to such strategy alterations during ~~the~~ performance~~, is in~~ . We also confirmed our ~~opinion,~~hypothesis that the LOS test might replace FR test in the diagnosis of ESs, which leads to conclusion that among healthy ESs the computerized posturography should be considered a more appropriate ~~to~~measure for the early identification of balance deficits than subjective tests and FR test.

Other clinical balance test scores as well as FR test are poor predictors of the fear of falling in groups of healthy ~~ES~~ESs. In the present study, only ~~the~~ TUG test were correlated with ~~the~~ FESI scores. Neither of them were correlated with a history of falls. Landers et al. ~~2015 (27~~(35) showed which self-reported questionnaires are most clinically applicable to the prediction of falls in the elderly population. They also indicated that ~~the~~ TUG test is the most predictive clinical test of the risk of falls. In general, clinical tests detect visible balance deficits. However, ~~an~~the identification of postural control changes that are invisible to ~~a~~the naked eye should be pursued. The FFSI allows ~~not only to detect~~ the detection of the area of the safety margin, ~~but~~ ~~also~~and it is correlated with the FOF in healthy ~~ES~~ESs. Moreover, the FFSI is also highly correlated with clinical test scores, such ~~a~~as TUG~~, Tinneti~~ and Tinetti test scores, which are known to be good predictors of the risk of falls (~~28~~2). We can conclude that, based on our results, it is not necessary to use the whole battery of clinical tests for balance assessment ~~and~~ ~~still~~to achieve satisfactory diagnostic outcomes~~.~~, and our research hypothesis was validated. Acquiring more information in less time is especially important when conducting ~~diagnosis~~diagnoses in specific populations and situations.

D~~iagnosis~~The diagnosis of the PD subjects led to different observations. According to the Hoehn and Yahr ~~Scale~~scale, all PD subjects in this study were in stage III ~~state~~. Everyone had ~~a~~ visible and persistent changes in postural control (~~2~~3), which could be detected by both clinical tests and posturography measurements. All clinical test scores were highly correlated with the FOF. Additionally, the Tinetti and TUG test were highly correlated with a history of falls. Nevertheless, the FFSI had a poor correlation with the FOF, but ~~it is~~was highly correlated with a history of falls. Moreover, the FFSI was associated with only ~~with~~ two clinical tests of the risk of falls. ~~Therefore,~~Our results validated our research hypothesis; therefore we postulate that ~~the~~ balance ~~assessment~~assessments of the PD population should be made both with ~~the~~ clinical ~~test~~tests and ~~the~~with computerized posturography. Clinical tests also assess movements such as walking forward, quiet standing and sitting down, which are indicated by Robinovitch et al. (36) as the most risk of falling activities.

As ~~it occurred~~shown, the differences between PD subjects and their healthy peers can be easily detected with BBS and TUG ~~test. Although~~tests. Whereas the TUG test is an incontestably appropriate measure to assess the dynamic balance among PD subjects, using the BBS for dynamic balance assessment is more questionable (~~29~~37). Some authors indicated that the BBS, when used to measure balance in patients with PD, may not completely assess the whole spectrum of postural control impairments typical for this disease (~~30~~38). Moreover, La Porta et al. (~~2015) (31~~39) suggested that the BBS, even if modified by the new rating scale, may not be an effective tool for the measurement of early postural control disturbance in patients with PD. Although there were no differences between PD subjects and the control group, it could be observed that the LOS test was more demanding for PD subjects. During the 3rd phase of the test, where subjects had to maintain an inclined position, PD subjects were characterized by lower values of ~~Sample entropy.~~SampEn. According to ~~Richmanand~~Richman and Moorman (2000) (24), low values of entropy show more regularity in the COP signal. ~~Thereby~~Therefore, less chaotic excursions may be defined as a characteristic of an unsuccessful vigilant strategy to ~~keep~~maintain balance. Furthermore, Donker et al. (~~2008) (32~~40) showed that the amount of attention invested in ~~keepingan~~keeping an upright ~~posturee~~posture is positively correlated with sway regularity. Based on these ~~report~~reports, PD subjects paid more attention to ~~keeping~~ maintaining an inclined position than the control group (fig~~. 2).~~ 1). Therefore, we confirmed our research hypothesis that the LOS test is a more challenging motor task for PD subjects. Reinert et al. (~~2017) (33~~41) confirmed that the use of nonlinear analysis methods such as ~~sample~~ ~~entropy~~SampleEn may improve the ability of the LOS test to identify differences in performance that are not currently revealed by traditional sway ranges.

This study has several limitations. A larger sample size would have been better as it would help to better generalize the results. Additionally, it is known that psychological factors, which had not been taken into account in this study, are associated with ~~the~~ increased postural threat ~~also~~and could also affect the processing of postural control. Finally, it is known that pharmacological treatments may have an impact on postural control in PD ~~patients~~subjects. Although we did our best to satisfy the standardization of procedures, we did not explore the problem of the impact of medication on postural control. Future research should be focused on the prospective risk of falls. It will be useful to create FFSI norms that can help diagnose the risk of falls.

## Conclusion

The present study highlights the importance of a quantitative approach for balance evaluation in ~~ES~~ESs and ~~also~~ elderly subjects with ~~a~~ neurological disorders. ~~Functional~~The functional forward stability indicator is a good predictor of the fear of falling in the group of elderly people. Additionally, ~~it~~the FFSI allows us to show real balance deficits both in PD ~~patients~~subjects and in their healthy peers without using an external reference group or created norms. A complete assessment of postural balance in PD ~~patients~~subjects should be made both with posturography ~~measurement~~measurements and with clinical ~~test~~tests.

**~~Acknowledgements~~**

~~The study was supported by the~~ ~~National~~ ~~Center for Research and Development~~ ~~Grant under~~

~~the	program	STRATEG~~	~~MED	III~~	~~within	the	“VB-Clinic”	project	no.~~

~~STRATEGMED3/306011/1/NCBR/2017.~~

# Conflict of interest statement

All authors confirm that there are no known conflicts of interest.

# Bibliography

1.	Haagsma JA, Olij BF, Majdan M, Beeck EF Van, Vos T, Castle CD, et al. Falls in older aged adults in 22 European countries : incidence , mortality and burden of disease from 1990 to 2017. Inj Prev. 2020;1–8.

~~2~~2.	Ambrose AF, Paul G, Hausdorff JM. Risk factors for falls among older adults: A review of the literature. Maturitas [Internet]. 2013;75(1):51–61. Available from: http://dx.doi.org/10.1016/j.maturitas.2013.02.009

3.	Lee HK, Altmann LJP, McFarland N, Hass CJ. The relationship between balance confidence and control in individuals with Parkinson’s disease. Park Relat Disord.

2016;26:24–8.

~~3~~4.	Tangen GG, Bergland A, Engedal K, Mengshoel AM. The importance of parkinsonian signs for gait and balance in patients with Alzheimer’s disease of mild degree. Gait Posture ~~.~~[Internet]. 2017;51:159–61. Available from: http://dx.doi.org/10.1016/j.gaitpost.2016.10.009

~~4~~5.	Stolze H, Klebe S, Zechlin C, Baecker C, Friege L, Deuschl G. Falls in frequent neurological diseases: Prevalence, risk factors and aetiology. J Neurol.

2004;251(1):79–84.

~~5~~6.	Park JH, Cho H, Shin JH, Kim T, Park SB, Choi BY, et al. Relationship among fear of falling, physical performance, and physical characteristics of the rural elderly. Am J Phys Med Rehabil. 2014;93(5):379–86.

~~6~~7.	Del-Río-Valeiras M, Gayoso-Diz P, Santos-Pérez S, Rossi-Izquierdo M, FaraldoGarcía A, Vaamonde-Sánchez-Andrade I, et al. Is there a relationship between short FES-I test scores and objective assessment of balance in the older people with ageinduced instability? Arch Gerontol Geriatr~~.~~ [Internet]. 2016;62:90–6. Available from: http://dx.doi.org/10.1016/j.archger.2015.09.005

~~7~~8.	Kempen GIJM, Todd CJ, Van Haastregt JCM, Zijlstra GAR, Beyer N, Freiberger E, et al. Cross-cultural validation of the Falls Efficacy Scale International (FES-I) in older people: Results from Germany, the Netherlands and the UK were satisfactory. Disabil Rehabil. 2007;29(2):155–62.

~~8~~9.	Maki BE, McIlroy WE. Control of rapid limb movements for balance recovery: Agerelated changes and implications for fall prevention. Age Ageing.

2006;35(SUPPL.2):12–8.

~~9~~10. Forth KE, Fiedler MJ, Paloski WH. Gait & Posture Estimating functional stability boundaries for bipedal stance. Gait Posture ~~.~~[Internet]. 2011;33(4):715–7. Available from: http://dx.doi.org/10.1016/j.gaitpost.2010.12.024

~~10~~11. Błaszczyk J.W., Lowe D. L. HPD. Ranges of postural stability and their changes in the elderly. Gait Posture. 1994;2~~:~~(November):11–7.

~~11.	Chan~~12.	CHAN CW, ~~Rudnis~~RUDINS A. Foot Biomechanics During Walking

and Running. Mayo Clin Proc~~.~~ [Internet]. 1994;69(5):448–61. Available from: http://dx.doi.org/10.1016/S0025-6196(12)61642-5

~~12~~13. O’Neill TW, Varlow J, Silman AJ, Reeve J, Reid DM, Todd C, et al. Age and sex influences on fall characteristics. Ann Rheum Dis. 1994;53(11):773–5.

14. Youn J, Okuma Y, Hwang M, Kim D, Cho JW. Falling Direction can Predict the

Mechanism of Recurrent Falls in Advanced Parkinson’s Disease. Sci Rep.

2017;7(1):7–12.

15. Duarte M, Zatsiorsky VM. Effects of body lean and visual information on the equilibrium main- tenance during stance. Exp Brain Res. 2002;60–9.

~~13~~16. Pauelsen M, Vikman I, Johansson Strandkvist V, Larsson A, Röijezon U. Decline in sensorimotor systems explains reduced falls self-efficacy. J Electromyogr Kinesiol~~.~~

[Internet]. 2018;42(May):104–10. Available from:

https://doi.org/10.1016/j.jelekin.2018.07.001

~~14~~17. Kamieniarz A, Michalska J, Brachman A, Pawłowski M, Słomka KJ, Juras G. A posturographic procedure assessing balance disorders in parkinson’s disease: A systematic review. Clin Interv Aging. 2018;13:2301–16.

~~15~~18. Cleworth TW, Inglis JT, Carpenter MG. Postural threat influences the conscious perception of body position during voluntary leaning. Gait Posture [Internet].

2018;66(April):21–5. Available from: https://doi.org/10.1016/j.gaitpost.2018.08.003

19. Johansson J, Jarocka E, Westling G, Nordström A, Nordström P. Predicting incident falls: Relationship between postural sway and limits of stability in older adults. Hum

Mov Sci [Internet]. 2019;66(October 2018):117–23. Available from: https://doi.org/10.1016/j.humov.2019.04.004

20. Stins JF, Michielsen ME, Roerdink M, Beek PJ. Sway regularity reflects attentional involvement in postural control: effects of expertise, vision and cognition. Gait Posture [Internet]. 2009 Jul [cited 2015 Apr 28];30(1):106–9. Available from: http://www.ncbi.nlm.nih.gov/pubmed/19411174
21. Roerdink M, Hlavackova P, Vuillerme N. Center-of-pressure regularity as a marker for attentional investment in postural control: a comparison between sitting and standing postures. Hum Mov Sci [Internet]. 2011 Apr [cited 2013 Aug 23];30(2):203–12.

Available from: http://www.ncbi.nlm.nih.gov/pubmed/20542347

22. Jean-Baptiste Mignardot, Olivier Beauchet CACC, Deschamps T. Postural sway, falls, and cognitive status: a cross-sectional study among older adults. J Alzheimers Dis.

2014;41(2):431–9.

~~16~~23. Juras G, Słomka K, Fredyk A, Sobota G, Bacik B. Evaluation of the Limits of Stability (LOS) Balance Test. J Hum Kinet. 2008;19(1):39–52.

~~17~~24. Richman JS, Moorman JR. Physiological time-series analysis using approximate entropy and sample entropy. AmJ Physiol Hear CircPhysiol. 2000;278~~:~~(0363-6135 (Print)):H2039–49.

~~18~~25. Ratner B. The correlation coefficient: Its values range between 1/1, or do they. J

Targeting, Meas Anal Mark. 2009;17(2):139–42.

~~19~~26. Portney LG WM. Foundations of Clinical Research: Applications to Practice. Upper

Saddle River, NJ: Prentice Hall Health; 2000.

27.	Clark S, Iltis PW, Anthony CJ, Toews A. Comparison of Older Adult Performance

During the Functional-Reach and Limits-of-Stability Tests. J Aging Phys Act. 2005;13:266–75.

~~20~~28. Ryckewaert G, Luyat M, Rambour M, Tard C, Noël M, Defebvre L, et al. Selfperceived and actual ability in the functional reach test in patients with Parkinson’s disease. Neurosci Lett [Internet]. 2015;589:181–4. Available from:

http://linkinghub.elsevier.com/retrieve/pii/S0304394015000440

29.	Mancini M, Rocchi L, Horak FB, Chiari L. Effects of Parkinson’s disease and

levodopa on functional limits of stability. Clin Biomech. 2008 May;23(4):450–8.

~~21~~30. Behrman AL, Light KE, Flynn SM, Thigpen MT. Is the functional reach test useful for identifying falls risk among individuals with Parkinson’s disease? Arch Phys Med Rehabil. 2002;83(4):538–42.

~~22~~31. Słomka KJ, Michalska J, Marszałek W, Bacik B, Juras G. Forward functional stability

indicator (FFSI) as a reliable measure of limits of stability. MethodsX. 2020;7:10–6.

~~23~~32. Lesinski M, ~~Hortobagyi~~Hortob??gyi T, Muehlbauer T, Gollhofer A, Granacher U. Effects of Balance Training on Balance Performance in Healthy Older Adults: A Systematic Review and Meta-analysis. Sport Med. 2015;45(12):1721–38.

~~24~~33. Horak FB. Postural orientation and equilibrium: What do we need to know about neural control of balance to prevent falls? Age Ageing. 2006;35(SUPPL.2):7–11.

~~25~~34. Horak F, Kuo A. Postural adaptation for altered environments, tasks, and intentions. In:

Biomechanics and neural control of posture and movement. Springer; 2000. p. 267–81.

~~26~~35. Kage H, Okuda M, Nakamura I, Kunitsugu I, Sugiyama S, Hobara T. Measuring Methods for Functional Reach Test: Comparison of 1-Arm Reach and 2-Arm Reach.

Arch Phys Med Rehabil~~.~~ [Internet]. 2009;90(12):2103–7. Available from:

http://dx.doi.org/10.1016/j.apmr.2009.07.021

~~27~~36. Landers MR, Oscar S, Sasaoka J, Vaughn K. Balance Confidence and Fear of Falling Avoidance Behavior Are Most Predictive of Falling in Older Adults: Prospective

Analysis. Phys Ther. 2015;96(4):433–42.

~~28~~37. Robinovitch SN, Feldman F, Yang Y, Schonnop R, Leung PM, Sarraf T, et al. Video capture of the circumstances of falls in elderly people residing in long-term care : an observational study. 2010;47–54.

38.	Ambrose AF, Paul G, Hausdorff JM. Risk factors for falls among older adults: A review of the literature. ~~Maturitas. 2013;75(1):51–61. Available from:~~

~~29~~.	Boulgarides LK, Mcginty SM, Willett JA, Barnes CW. Use of Clinical and Impairment-Based Tests to Predict Falls by Community-Dwelling Older Adults. Phys Ther. 2003;83(4):328–39.

~~30~~39. King LA, Priest KC, Salarian A, Pierce D, Horak FB. Comparing the Mini-BESTest with the Berg Balance Scale to evaluate balance disorders in Parkinson’s disease.

Parkinsons Dis. 2012;2012.

~~31~~40. La Porta F, Giordano A, Caselli S, Foti C FF. Is the Berg Balance Scale an effective tool impairments in patients with Parkinson’s disease? for the measurement of early postural control Evidence from Rasch analysis. Eur J Phys Rehabil Med. 2015;51:705–

16.

~~32~~41. Donker SF, Ledebt A, Roerdink M, Savelsbergh GJP, Beek PJ. Children with cerebral palsy exhibit greater and more regular postural sway than typically developing children. Exp Brain Res. 2008;184(3):363–70.

~~33~~42. Reinert SS, Kinney AL, Jackson K, Diestelkamp W, Bigelow K. Age stratification and sample entropy analysis enhance the limits of stability tests for older adults. J Appl

Biomech. 2017;33(6):419–23.

**Supporting information**

**S1 Fig 1 The median value of sample entropy in the anterior-posterior direction**

**(minimum and maximum marked as error bars) in the first and third phases of the limit**

**of stability test.**

**S2 Fig 2 Forward stability region.**

**S1 Table 1 Characteristics of subjects**

**S2 Table 2 Spearman’s correlation coefficient between FFSI and balance clinical test in**

**ESs and PD subjects**

**S3 Table 3 Spearman’s correlation coefficients between among balance clinical test scores, the FES-I score, and the number of falls in elderly subjects and PD subjects**

550

Response to Reviewers

**Editor comment:**

1. **Please ensure that your manuscript meets PLOS ONE's style requirements, including those for file naming.**

**Answer:**

In the current revision we have followed all the PLOS ONE style requirements including those for file naming.

2. **Please provide additional details regarding participant consent. In the ethics statement in the Methods and online submission information, please ensure that you have specified (1) whether consent was informed and (2) what type you obtained (for instance, written or verbal, and if verbal, how it was documented and witnessed). If your study included minors, state whether you obtained consent from parents or guardians. If the need for consent was waived by the ethics committee, please include this information.**

**Answer:**

The additional details regarding participant consent were provided. The following sentences were added to the methods section: “The subjects gave informed written consent for voluntary participation in the study. The research was approved by the Institutional Ethics Committee of the Medical University of Warsaw (number KB/28/2014).”

3. **Please ensure that you refer to Figure 1 in your text as, if accepted, production will need this reference to link the reader to the figure.**

We have reformatted the equation in Word’s “Equation Tools” and removed equation as a figure form current manuscript. It is now referred to as an equation.

4. **We note you have included a table to which you do not refer in the text of your manuscript. Please ensure that you refer to Table 1 in your text; if accepted, production will need this reference to link the reader to the Table.**

**Answer:**

The reference in the text to Table 1 was added: …“Sixteen PD subjects and sixteen anthropometrically matched healthy controls voluntarily participated in the study (Table 1).”

5. **Please include captions for your Supporting Information files at the end of your manuscript, and update any in-text citations to match accordingly.**

We have added Supporting Information files at the end of the manuscript.

**Reviewer #1**

***Dear authors, Thank you for your submission and your contribution to the field. This study is interesting with some worthy outcomes. I have attached a copy of your submission with some comments and suggestions. In summary, my main reason for recommending a major review is the lack of focus in the paper and also some paragraphs are difficult to understand. I suggest to review what precise questions you are asking of the data, clearly formulate hypotheses based on previous research and stick with these tightly throughout the paper. Points are made in the introduction but especially the discussion that are not directly related to the questions asked in this paper and can be shortened or removed. Overall it is a good paper and after some changes will be ready for publication.***

Answer:

First of all, we would like to thank the reviewer for a very insightful review. The manuscript was amended thoroughly according to the reviewer’s comments and suggestions. Some issues have risen after reading the review, which is explained in detail below. Referring to the reviewer's concerns about language, we have sent our manuscript to The American Journal Experts (the certificate was attached). So all the proofread suggestions have been done.

***Reviewer comment***

***The paragraph (lines 55-59/after correction lines 55-59) is not very clear to me..I understand that increased fear of falling may be a reaction to physical/cognitive deteriorations that can lead to beneficial cautious behaviour but it can also exacerbate the risk of falling.***

Answer:

To clarify this sentence we have provided changes which are the following:

Lines 51-56: “The fear of falling (FOF) could appear well before and/or after an incident of fall which creates a vicious cycle. Concomitant psychological symptom of falls is the fear of falling, which is common among older adults whether or not they have sustained a fall. FOF might also be a proper reaction to certain situations, leading the elderly to be cautious behaviour, and can contribute to fall prevention through careful choice of movement activity. Nevertheless, ESs increasingly cease physical activity because of the FOF (5).”

***Reviewer comment***

***Lines 80-86: This problem statement needs to be made clearer .I believe you wish to compliment the disadvantages of clinical measures with more objective measures of stability however this is not directly stated.***

Answer:

We have added following sentence to clarify the problem:” Clinical tests are often based on a qualitative assessment (BBS, Tinetti test). Despite the clear assessment instructions, the researcher may influence the test results.”

***Reviewer comment***

***Your aim is not specifically addressing the problem statement in the previous paragraph… too general…perhaps with the aim of finding objective predictors of fall risk that can support/replace clinical assessments while also being specific to the patient needs is what you are aiming at? ..sample entro-py is also not described.***

Answer:

We agree with the reviewer, that the aim of our study is too general, so we have changed this paragraph as follows:

Lines: 101- 111 - “The aim of this study was to determine an objective predictor of the fear of falling and falls in ESs and patients with neurodegenerative disorders. For this purpose, the clinical and laboratory tests were analyzed. Depending on the study groups, the proposed objective measure could replace or support clinical assessment. Posturographic examination can also register early and nonvisible postural control changes, so we assumed that this objective assesment might replace clinical tests among ESs. We also hypothesized that the identification of the real functional limit of support with the LOS test would allow us to assess not only the fear of falling but also the risk of falls. Neurological disorders lead to changes in the mechanism of postural control (4). We assumed that PD patients present different balance strategies when their COG is located near their stability boundary. The analysis of the sample entropy should allow us to identify these changes.”

We believe that these corrections would facilitate reading and understanding the discussion.

***Reviewer comment***

***Include number of males and females in each group.***

Answer:

This information has been added in table 1. There were 9 males and 7 females in the PD group, and 8 males and 8 females in control group.

***Reviewer comment***

***How did you ensure there was no significant cognitive impairment in the PD sample?***

Answer:

PD subjects had the results of the Mini Mental State Examination (MMSE) in their medical history. All of them obtained more than 26 points. We have added this information in eligibility criteria as follows: “… no consent to participate in the research; dementia or cognitive impairment (MMSE score below

26 points); and neuromuscular, vestibular or orthopedic disorders “

***Reviewer comment***

***Summary statistics for all measurements should be presented, whether they are significant or not…not just sample entropy.***

Answer:

All results have been presented in the manuscript – significant or not. The reviewer might have not noticed this fact because in order not to duplicate the data presentation the results of the comparison between groups are located in the main text of the manuscript (FFSI, sampEn) and in table 1 (FES-I, BBS, FR, Tinetti test, TUG). We described the results of FFSI and SampEn in details, because we built our conclusion based on these observations. All correlations are presented in table 2 and 3.

***Reviewer comment***

***A summary of the findings in your study and if they met or did not meet your hypotheses would be good to start with.***

Answer:

Thank you for this comment. Following this suggestion we have pointed our hypothesis in our discussion as follows:

1) Line 256-257: “…So the existence of a safety margin might indicate deficits in balance, thus our research hypothesis was validated…”
2) Line 292-295: “…We can conclude that, based on our results, it is not necessary to use the whole battery of clinical tests for balance assessment to achieve satisfactory diagnostic outcomes, and our research hypothesis was validated.…”
3) Line 304-306 “…Our results validated our research hypothesis; therefore we postulate that balance assessments of the PD population should be made both with clinical tests and with computerized posturography …”
4) Line 326-327: “…Therefore, we confirmed our research hypothesis that the LOS test is a more challenging motor task for PD patients …”

***Reviewer comment***

***This paragraph (221-233/after correction 252-266) needs to be made clearer… there are many points being made that are not aligned, making it difficult to read.***

Answer:

Thank you for pointing this out. We have revised this paragraph and made all necessary grammar and spelling corrections. We believe that it is now more clear. We have also edited and added following sentences: “A greater safety margin decreases the range of the maximum forward lean, thereby decreasing functional movement. So the existence of a safety margin might indicate deficits in balance…”

***Reviewer comment***

***Figure 3 should be in the methods in my opinion***

Answer:

We appreciate this comment, however figure 3 (after correction figure 2) already presents some of results , therefore we put it in the discussion section.

***Reviewer comment***

***Line: 239-252(after corrections lines:272-282) Much of this can be summarized in a smaller number of sentences***

Thank you for this comment. The paragraph was revised and made more concise for the sake of clarity and readability***.*** The number of sentences was reduced.

***Reviewer comment***

***Line: 265-267 (after corrections lines 279-282)“Therefore, the LOS test being less prone to such strategy alterations during the performance, is in our opinion, more appropriate to early identification of balance deficits” – evidenced or opinion?***

Answer:

We fully understand reviewer’s concern. We have changed our hypothesis as follow: Depending on the study groups, the proposed objective measure could replace or support clinical assessment. Posturographic examination can also register early and nonvisible postural control changes, so we assumed that this objective assessment might replace clinical tests among ESs.” Therefore, we have presented our opinion based on the results.

***Reviewer comment***

***Line 277-280 (after correction lines 292-296) “We can conclude that based our results, it is not necessary to use the whole battery of clinical tests for balance assessment and still achieve satisfactory diagnostic outcomes. Acquiring more information in less time is especially important when conducting diagnosis in specific populations and situations.” – was this an aim?***

Answer:

Once again we fully understand reviewer’s concern. We have changed our hypothesis, so statement in the current form correspond to the aim of our study.

**Reviewer #2**

***Introduction***

***Reviewer comment 1:***

***General – language. For publication in PLOS ONE, the article should be “presented in an intelligible fashion and is written in standard English”. The authors need to spell check and go through their manuscript. Getting language editing help is recommended.***

Answer:

At the beginning, we would like to express thanks to the reviewer for a very insightful review. The manuscript was corrected according to the reviewer’s suggestions. Your comments have contributed to improvement our paper. The manuscript was proofread by a certified proofread agency (certificate attached in the files).

***Reviewer comment 2:***

***Why is there no citation after “The ES experience falls due to significant deterioration of balance ability associated with decrease of muscle strength, sensory function and widespread degeneration in the central nervous system.”?***

Answer:

Thank you for pointing this out. We have added missing citation: Ambrose AF, Paul G, Hausdorff JM. Risk factors for falls among older adults: A review of the literature. Maturitas.2013; 75 (1): 51-61.

***Reviewer comment 3***

***Please revise the unclear sentence “The questionnaire refers to the most of the basic, everyday activities presented by disabled people would be likely to have difficulty with because of their FOF. :***

Answer:

We have revised this sentence:“The questionnaire refers to the most of the basic, everyday activities. Only people with FOF may have difficulty to perform these actions.”

***Reviewer comment 4***

***Limit of Stability is not abbreviated at the first time of use.***

Answer:

Thank you for pointing this out. We have abbreviated Limit of Stability (LOS) at the first time of use .

***Reviewer comment 5***

***The calculation of maximum forward lean range with respect to the mechanical (anatomical) stability boundary should allow to demonstrate the real safety margin. It is crucial for appropriate assessment of fear and risk of falling.” – Says who? Says you? Nothing in this sentence supports just using forward lean range. Medio-lateral leaning and LOS could be meaningful as well! Please revise and use references when building argumentation. Also, you can never be sure that you reach the “real” safety margin, as participants may stop themselves from leaning as far as they possibly can out of fear of falling or reduced joint flexibility. It is very difficult to achieve the “real” safety margin. I suggest you rephrase to something like “ should allow us to reach the boundary that is in close proximity to the real safety margin”***

Answer:

Thank you for rising this issue. To clarify why we focused our research only on the forward stability boundary, we have added the following sentences to the introduction: “In older adults over 65 yr, 60% of falls occur in a forward direction (O’Neill et al. 1994). Additionally, Youn et al. (2017) noticed that more than 70% of PD patients had a history of falls in the forward direction. Therefore, in the present study, we focused on exploring the anterior stability boundary”. We also agree with the reviewer, that we can never be sure that we reach the “real” safety margin, as participants may stop themselves from leaning as far as they possibly can out of the fear of falling or reduced joint flexibility. Therefore, we have rephrased this sentence according to the reviewer’s suggestion: “The calculation of the maximum forward lean range with respect to the mechanical (anatomical) stability boundary should allow subjects to reach the boundary that is in close proximity to the real safety margin”. We also have added references: Błaszczyk J.W., Lowe D. Ranges of postural stability and their changes in the elderly. Gait & Posture 1994(2):11-7. The authors have pointed out the importance of the detection of safety margin: “Reduction of the maximum voluntary excursion increases the probability of recovery from postural instability by increasing the margin safety, which allows for more time to complete the recovery programme”.

***Reviewer comment 6***

***“Since falls usually occur during movements where the COG is shifted near or outside BOS, it seems that the LOS test, is the most appropriate measurement.” – Again, no references to support this statement. Also if falls usually occur during movements, is this really associated with a LOS test where a participants stands bilaterally, relatively static, and leans in different directions? Shouldn’t it rather be associated with people walking, stepping and how the COG moves within the LOS then?***

Answer:

We agree that this statement should be supported by proper reference. However, this statement was created based on knowledge about postural strategy. Upright posture is controlled during disturbance by the ankle and hip joint movements, classified as the “ankle strategy” and “hip strategy”, respectively. The greater the postural disturbance, the closer the COG moves to the stability boundary. When the COG crosses the stability boundary, the subject has to take a step to recover the upright posture. According to Clark et al. (2005), for many older adults, spatial and temporal control of the COG is decreased, and movement strategies become unwieldy, resulting in an increased risk of falls when performing reaching tasks or gait initiation. Therefore, we have rephrased the sentence as follows: “With an inefficient postural strategy (ankle and hip strategy) in elderly individuals, falls usually occur during movements where the COG is shifted near or outside the BOS. It seems that the LOS test is the most appropriate measurement.” We have also added the appropriate reference (Cleworth et al. 2018 Gait & Posture).

***Methods:***

***Reviewer comment 1***

***The recruitment and inclusion procedure is not described thoroughly. It says that sixteen PD subjects and sixteen age-matched healthy controls voluntarily participated. Was this a convenience sample? How did recruitment proceed? Was this done at a hospital? Were the controls matched only on age? How will eventual non-matched variables confound your results?***

Answer:

Again, thank you for pointing this out. We have provided several corrections in the description of this section. We recruited PD subjects from the Local Association of Parkinson's disease community. We have changed the phrase from “PD patients” on “PD subjects”. One of our co-authors, who is the specialist of PD physiotherapy verified the inclusion and exclusion criteria. Subjects who met eligibility criteria were invited to examination. The research took place at the Academy of Physical Education in Katowice (Poland). The sample size of PD subjects was determined by the number of subjects from the Association of Parkinson's Disease who met the eligibility criteria. The control group was matched not only for age, but also for body height and weight. Table 1 presents the anthropometric values of the two groups. All of the above details have been included in the manuscript.

***Reviewer comment 2***

***Please indicate how many men and women there were***

Answer:

Thank you for this comment. This information was added to the manuscript (table 1). In the PD group there were 9 males and 7 females, in control group there were 8 males and 8 females.

***Reviewer comment 3***

***The research was accepted by the Institutional Bioethics Committee.” – Where is this committee situated? More details required.***

Answer:

We have changed the sentence as follow: “The research was accepted by the Institutional Ethics Committee Medical University in Warsaw number KB/28/2014”.

***Reviewer comment 4***

***Table 1 – p-values are given but can be misinformative when there is risk for low statistical power. I encourage the authors to present confidence intervals instead. Also, the statistic section says that normal distribution was controlled for, but there is no information whether the parameters were normally distributed or not. Also, the “independent-samples t-test was conducted”. Did you use a parametric or non-parametric t-test?***

Answer:

We agree that the section of statistical methods should be written with more details. Depending on the variable distribution, parametric or nonparametric statistical analysis was used. All anthropometric variables, the FFSI and the SampEn, were normally distributed compared to FES-I, BBS, TUG, FR and Tinetti test scores; therefore, we used parametric and nonparametric tests, respectively. To compare values of age, weight, body weight, the FFSI and the SampEn between groups, an independent-sample t-test was conducted. To compare FES-I, BBS, TUG, FR and Tinetti test scores, the Mann-Whitney U test was conducted. All correlations were calculated by the nonparametric Spearman rank test. We have corrected this section as follows: “The parametric and nonparametric tests were used. An independent-sample t-test was conducted to compare the anthropometric variables FFSI and Samp En from ESs and PD patients. The Mann-Whitney U test was conducted to compare FES-I, BBS, Tinetti, FR and TUG test scores from ESs and PD patients.” We have removed p-values from table one and we have added confidence intervals.

***Reviewer comment 5***

***Table 1 – I suggest putting percentages after the values on low-moderate-high concern to make it clearer.***

Answer:

We have put percentages after the values on low-moderate-high concern in table 1.

***Reviewer comment 6***

***Table 1 – for BBS, FR, Tinetti test and TUG; indicate what unit is presented (score, seconds, etc.), e.g. “TUG (s)”***

Answer:

Table 1 was revised according to reviewers comments.

***Reviewer comment 7***

***From the methods it becomes clear that the study investigates fall history and not prospective falls. This should be indicated in the manuscript title as “history of falls”. This means that the outcome is prevalent before the risk factor is measured, and could likely influence the results. This should be mentioned in the limitation section.***

Answer:

Once again, thank you for pointing this out. We have changed the title for: ” Fall-related measures in elderly individuals and Parkinson’s disease subjects”. We also added to the limitation section following sentences: “Future research should be focused on prospective risk of falls. Future research should be focused on the prospective risk of falls. It will be useful to create FFSI norms that can help diagnose the risk of falls.”

***Reviewer comment 8***

***The forward leaning LOS test is used. The advantages and disadvantages of this test needs to be discussed more. What information on LOS is missed? The authors early cite Forth et al. 2011 that in their paper states “However, an individual’s functional stability boundary during bipedal stance is more likely an ellipse much smaller than the base of support” The forward lean LOS test does not cover the other directions in an ellipse, and falls can occur in several directions. If the manuscript should be placed in the context of fall prediction, the validity of the test, e.g. strength and weaknesses should be properly discussed.***

Answer:

Thank you for pointing this out, as we have mentioned before, we focused on forward stability boundary, because of the frequency of falls in this direction. We wrote about some advantages and disadvantage of the LOS test in section of discussion.

***Reviewer comment 9***

***“The LOS test lasted 30 seconds and was repeated three times.” – were all three values analyzed? Or did you use the maximal value? Or an average over the three? Please specify. If using an average – discuss if you think test fatigue might influence the results.***

Answer:

Thank you for this comment. We have added calculation of the intraclass correlation coefficients for the limit of stability test in the statistical section as follows: “The levels of correlation were considered weak positive (0 – 0,3), weak negative (-0,3 – 0), moderate positive (0,3 – 0,7), moderate negative (-0,3 – (-0,7)), high positive (0,7 – 1,0), and high negative (-0,7 – (-1,0)) (18). Intraclass correlation coefficients (ICCs) were calculated for the LOS test (FFSI, sampEn phase 1, sampEn phase 3). The levels of reliability were considered poor (ICC < 0.50), moderate (0.50 ≤ ICC < 0.75), good (0.75 ≤ ICC < 0.90), and excellent (ICC ≥ 0.90), according to Portney et al (2000). The LOS test achieved excellent reliability with 3 repetitions..” – this of course means that we took average of three trials for further analysis. The fatigue probably might influence the result of the test if there were not rest break between repetitions. That is why each research subjects were getting off the force plate after each trial and could rest.

***Reviewer comment 10***

***I suggest helping the reader understand the FFSI better by adding this parameter its unit of measure (%?) to Table 1***

Answer:

Thank you for this suggestion. We have added measurement units of FFSI to the Table 1.

***Results***

***Reviewer comment 1***

***Table 2 – what does the red numbers indicate? Please specify***

Answer:

Again, thank you for the comment. Indeed, the red color might be misleading. So we have changed red to bold indicating statistical significance. The description of this was added to the table’s legend.

***Reviewer comment 2***

***Table 3 – it is said to report “number and direction of falls”. Please specify, did you record in what direction the participants had fallen? And if so, this is not reported in table 3.***

Answer:

Thank you for pointing this out. It was a mistake. We didn’t examine the direction of the falls. So we have corrected the title of Table 3 as follows: “Spearman’s correlation coefficients between among balance clinical test scores, the FES-I score, and the number of falls in elderly subjects and PD subjects.

***Discussion***

***Reviewer comment 1***

***Line 235-236 “Computerized posturography should be considered a more appropriate measure of balance ability among healthy ES than subjective tests.” What about the fact that most objective postural stability measures are performed in a static stance, while clinical tests are more dynamic in their nature? And falls often occur during movement and everyday situations, and not necessarily when standing still – for example, see Robinovitch et al. 2013 in the Lancet that have video-recorded falls.***

Answer:

We fully understand the reviewer’s concern. We agree that most objective measures are performed in a static stance, but falls predominantly occur during more dynamic motor tasks. Therefore, we chose the limit of stability test rather than quiet standing. Almost 30 years ago, Duncan et al. (1990) created a simple measure such as the Functional Reach Test, which was highly correlated with the risk of falls. Over the years, it turns out that indications of the functional limit of stability are crucial to assess the risk of falls. Even simple motor tasks such as body leaning on the force plate are very informative. We know exactly where the COP is located within the base of support. This allows for the calculation of the safety margin. No clinical test allows this. Additionally, having a physiotherapist’s experience, one can notice that the researcher has a large influence on the BBS and Tinetti test results.

***Reviewer comment 2***

***Line: 254-255 “However, an identification of postural control changes that are invisible to a naked eye should be pursued.”***

***Line 258-260 “We can conclude that based our results, it is not necessary to use the whole battery of clinical tests for balance assessment and still achieve satisfactory diagnostic outcomes.”***

***These statements require a better rationale. Why should we use objective measures? For fall risk? Can you really be sure this is better? There is evidence that self-reported fall history is a better predictor of prospective falls than objective tests, see for example Johansson et al. 2019 in Human Movement Science. So why should clinics spend resources on expensive equipment when it is better to ask a simple question?***

Answer:

We fully understand reviewer’s concern, however self-reported fall history assesses what happened in the past. We agree that, based on this report, research subjects have poor balance. However, is it possible to predict a fall before the first accident? The fear of falling can appear both before and after accidents or falls. In our study, we detected the fear of falling without a history of falls in almost all research subjects. Therefore, our indicator has great potential to determine the fear of falling. There is a high probability of detecting the risk of falling if the research is conducted with follow-up examinations. Thank you for suggesting Johansson et al. papers, we have added it to references

***Reviewer comment 3***

***Line: Conclusion on line 300-301 states “Functional forward stability indicator is a good predictor of fear of falling in group of elderly people.” – The manuscript should change its title to reflect this conclusion, so that it is about predictors of fear of falling rather than actual falls. The reader might misinterpret this and think that prospective falls are investigated***

Answer:

We completely agree with reviewer comment. So as we have mentioned before, we have changed the title of the manuscript as follows: “Fall related measures in elderly and Parkinson’s disease”.

***Reviewer comment 4***

***Line: Conclusion Line 224 “Existence of safety margin indicates deficits in balance.” – This singlesentence statement is unclear to me. Every human should have a safety margin, the question is how close to the safety margin our COG is displaced, and in turn might indicate deficits in balance.***

Answer:

We do agree with this logic. However, we wanted to present other ideas connected with functional efficiency. We agree that a greater safety margin indicates more time to introduce and choose the strategy of balance. However, a greater safety margin decreases the range of the maximum forward lean, thereby decreasing functional movement. All subjects should explore as much as possible of the base of support. In our understanding, the safety margin is located between the first metatarsal phalangeal joint and the location of the COP while keeping the body in a leaning position. If the COP is located closer to this joint, the safety margin has a smaller area. We hope that the reviewer would agree with this rationale.

***Reviewer comment 3***

***Also Line 224 “The greater safety margin is combined with greater postural instability (10).” If by safety margin you mean the margin between COG and LOS, this is not true for falls. Again, see Johansson et al. 2019 in Human Movement Science. Here, the greater the area of the center of pressure in relation to the LOS, the greater the risk for incident falls. This means that a greater safety margin was beneficial and reduced the fall risk.***

Answer:

In our study, smaller values of the range of maximum forward lean correlated with lower scores on some clinical tests (the BBS, FR test, and Tinetti test) and with higher scores on the TUG test. This clearly means that lower FFSI values indicate a greater risk of falling. Furthermore, a smaller range of maximum forward lean results in a greater safety margin

***Reviewer comment 3***

***Line 281 – please be consequent with your citation method. Here you have switched to writing out the names and the year, and have not used the numbered reference system used in the rest of the manuscript.***

Answer:

Thank you for pointing this out. All citations have been checked once again and we have made relevant correction.
