# Supplementary material for: Fall-related measures in elderly individuals and Parkinson’s disease subjects
Source: PLoS One. 2020 Aug 13;15(8):e0236886. doi: 10.1371/journal.pone.0236886 (PMC7425912; doi:10.1371/journal.pone.0236886)
Supplement: Attachment — Submitted filename: Response to Reviewers.docx [file pone.0236886.s005.docx]

**Reviewer #1**

***Reviewer comment*
*References required in this paragraph (line: 50-54).***

Answer:

Two references were added to support this paragraph: “Park et al. (2014) Am J Phys Med Rehabil.” and “Young et al. (2015) Gait Posture.”

***Reviewer comment***

***“Neurological disorders lead to changes in the mechanism of postural control.”- this statements requiring a reference should not be included in the aims paragraph as they should have already been stated previously and the aims and hypotheses should be self-evident.***

Answer:

Thank you for this comment. Following this suggestion we have removed this statement. We agree that statements requiring a reference should not be included in the aims paragraph. We wrote about the changes in the mechanism of postural control in PD subjects in the third paragraph of the introduction.

***Reviewer comment*
*Table 1 – Was a t-test or Mann Whitney U used? If so report the values so replication can be made possible.***

Answer:

Table 1 was revised. Depending on the distribution of variables, the values of t-test or Mann-Whitney U test were added to the table 1.

***Reviewer comment***

***Equation label required to differentiate it from figures***

Answer:

The equation label was added and incorporated in the text of the manuscript.

***Reviewer comment
I believe multiple comparisons requires a bonferroni correction to reduce type 1 error risk…for example by simply dividing 0.05 by the number of tests conducted for each hypotheses, one can arrive at the appropriate p value for significance…here it is 0.05 divided by 6 (the number of balance measures compared against the FFSI) giving you a p-value of 0.00833.***

Answer:

Thank you for raising this issue. The significance level had been revised and the Bonferroni correction was implemented (p = 0.0083) to reduce the possibility of statistical error. The information was added in the section of statistical:

„The Bonferroni correction was used to decrease risk of a type I error during making multiple statistical tests”.

We have also changed the indication of statistically significant on table 2.

***Reviewer comment
I believe Similarly, a Bonferroni correction will be required here also to reduce the risk of type 1 error..in this case it will need to be 0.05 divided by 8 as there is two dependent variables and two hypotheses giving you a p-value of 0.00625 for PD and ES subject comparisons..if you would like to avoid this correction it is best to reduce your analysis to an evidenced based search for specific correlations that are meaningful towards your overall aim..this could be carried out by selecting a smaller number of well-established balance assessments to compare against FFSI that address aspects of balance control individually….***

Answer:
Once again, thank you for this comment. As above, the significance level had been revised with a Bonferroni correction (p = 0.00625) to reduce the possibility of statistical error. We have provided new statistical significance in table 3.

***Reviewer comment***

***This first paragraph doesn’t address your aims, the purpose of the study, summary of findings or really anything relevant to your study except stating how the PD subjects were qualified as an at risk group for falls and how the measures were not sensitive..the reader should be reminded and brought into each section with a progressive building towards the conclusion rather than point statements about the findings***

Anwser:
Thank you for pointing this out. We have added a new paragraph in the discussion section as follows:

„The main aim of this study was to determine an objective predictor of the fear of falling and falls in ESs and PD subjects. That is way, we have analyzed both the qualitative and quantitative balance diagnostic methods, indicating that the posturographic examination can both register early and nonvisible postural control changes. Additionally LOS test assesses safety margin, as an alert of fear of falling. First, we assumed that the identification of the real functional limit of support surface with the LOS test would allow to assess not only the fear of falling but also the risk of falls. Second, we hypothesized that depending on the study groups, the proposed objective measure could replace or support clinical assessment. Third, we supposed that PD subjects present different balance strategies when their COG is located near their stability boundary.” We have also provided all hypothesis in the main text for discussion.

We would like to thank again the reviewer for a very insightful review. Your comments have contributed to improvement our paper.

**Reviewer #2**

***Reviewer comment* :
The authors' responses to my comments and questions are satisfactory. The rationale why they chose to investigate maximum forward lean (MFL) as a predictor for fear of falling is now clearer. LOS is a complex measure depending on how the postural stability within the stability boundary is assessed, and the authors have provided argumentation why the MFL can be relevant. Overall, the manuscript is clearer and linguistically improved, enhancing its readability. I have one additional comment that requires attention. It is not as severe as needing a 2nd round of review and can perhaps be checked by the editor:**

**Table 1 - confidence intervals for BBS (score) overlap, yet there is a bold marking for statistical significance. Please adjust accordingly, if it was significant and this is a typo or not.**

We are very grateful for a such an approving review. It strongly motivates us to do further research. There were no significant differences between ESs and PD subjects in BBS score. We have corrected a mistake in table 1.
